# Peripheral TIGIT+ T Follicular Helper Cells That Produce High Levels of Interleukin-21 *via* OX40 Represent Disease Activity in IgG4-Related Disease

**DOI:** 10.3389/fimmu.2021.651357

**Published:** 2021-04-14

**Authors:** Mitsuhiro Akiyama, Katsuya Suzuki, Keiko Yoshimoto, Hidekata Yasuoka, Yuko Kaneko, Tsutomu Takeuchi

**Affiliations:** ^1^ Division of Rheumatology, Department of Internal Medicine, Keio University School of Medicine, Tokyo, Japan; ^2^ Division of Rheumatology, Department of Internal Medicine, Fujita Health University School of Medicine, Aichi, Japan

**Keywords:** TIGIT, T follicular helper cells, interleukin-21, OX40, IgG4-related disease, disease activity, TPH

## Abstract

**Objectives:**

Multiple studies suggest that interleukin (IL)-21 plays a pivotal role in the differentiation of B cells and activation of cytotoxic T cells and is involved in the pathogenesis of IgG4-related disease (IgG4-RD). T cell immunoreceptor with immunoglobulin and ITIM domain (TIGIT) is a new marker of T follicular helper (Tfh) cells, yet its significance remains unknown. The objective of this study was to investigate whether TIGIT expression could detect high IL-21-producing peripheral Tfh populations and their association with disease activity in IgG4-RD.

**Methods:**

TIGIT expression in peripheral CD4+T cell subsets was comprehensively analyzed by multi-color flow cytometry. Single cell mapping was performed by t-SNE method, and IL-21 production was compared in TIGIT+ and TIGIT-T cells. The effect of OX40 signal on cytokine expression was analyzed by RNA-sequencing. Clinical significance of TIGIT+ and TIGIT- peripheral T cells was analyzed in active patients with IgG4-RD, both at baseline and after 12 weeks of glucocorticoid treatment.

**Results:**

Unbiased single cell mapping revealed two high IL-21-producing peripheral T cell populations; TIGIT+ Tfh and TIGIT-T helper cells. OX40 signal was associated with high IL-21 production in TIGIT+ Tfh and TIGIT-T helper cells. IL-21 production in Tfh cells correlated with the proportion of TIGIT+ cells in Tfh cells, serum IgG4 level, and scores of disease activity. Furthermore, the skewing toward peripheral TIGIT+ Tfh cells, particularly TIGIT+Tfh2 subset correlated with disease activity and was corrected by glucocorticoid treatment in IgG4-RD.

**Conclusions:**

OX40 is associated with high IL-21 production in peripheral TIGIT+ Tfh cells, and the increase in peripheral TIGIT+ Tfh cells reflects disease activity in IgG4-RD.

## Introduction

IgG4-related disease (IgG4-RD) is a fibro-inflammatory disease characterized by storiform fibrosis, IgG4+ plasma cell infiltration, and elevated levels of serum IgG4 (1–4). Mounting evidence has revealed that T follicular helper (Tfh) cells, which induce hyperplastic tertiary lymphoid organ formation, IgG4 class-switching, and plasma cell differentiation, are involved in the pathogenesis of IgG4-RD (5–15). In addition, CD4+ cytotoxic T cells cause tissue damage and fibrosis (16–19).

The expression of interleukin−21 (IL−21) is upregulated in the affected tissues of IgG4-RD patients (20). IL-21 is produced by CD4+ T cells, with high production by Tfh cells, and plays a key role in B cell differentiation and maturation and in enhancing the activity and survival of CD4+ cytotoxic T cells (21, 22). IL-21-producing Tfh cells thus may be a key immune cell subset involved in the pathogenesis of IgG4-RD.

Tfh cells in the peripheral blood of humans have been found to be CXCR5-positive, enabling their study (23, 24). Tfh cells can be divided into sub-populations according to their surface markers, including interferon-γ-producing Tfh1 (CXCR3+CCR6-), IL-4-producing Tfh2 (CXCR3-CCR6-) and IL-17-producing Tfh17 (CXCR3-CCR6+) (23, 24). Given that IL-21 plays a crucial role in the pathogenesis of IgG4-RD, we speculated that surface markers for IL-21-producing peripheral Tfh populations are likely surrogate markers for Tfh responses and may therefore be useful in the assessment of disease activity.

T cell immunoreceptor with immunoglobulin and ITIM domain (TIGIT) is a co-inhibitory receptor that is expressed on T cells and natural killer cells (25). Some studies have suggested that TIGIT expression can be detected on Tfh cells (26, 27). However, the usefulness of TIGIT expression as a surface marker of human peripheral Tfh cells remains unknown.

Here, we investigated the association of TIGIT with IL-21 expression in human peripheral Tfh cells. We also examined whether the proportion of peripheral TIGIT+ Tfh cells was associated with disease activity of IgG4-RD, a typical Tfh-associated autoimmune disease.

## Materials and Methods

### Patients and Methods

Twenty-three consecutive patients with untreated, active IgG4-RD (**Supplementary Table 1**), and 21 healthy individuals were included in this study. All patients with IgG4-RD fulfilled the 2011 comprehensive IgG4-RD diagnostic criteria or the 2019 American College of Rheumatology/European League Against Rheumatism classification criteria for IgG4-RD (28–30). Disease activity was assessed based on the IgG4-RD responder index (IgG4-RD RI) score (31). Healthy individuals were confirmed to have no autoimmune diseases, allergic disorders, malignancies, or infections. This study was approved by the ethics committee of Keio University School of Medicine and carried out in accordance with the Declaration of Helsinki. Written informed consent was obtained from all patients and healthy individuals.

### Immunophenotyping of Peripheral CD4+ T Cells and Measurement of IL-21 Production

To determine TIGIT expression on each CD4+ T cell subset, frozen PBMCs which were cryopreserved in liquid nitrogen were thawed first and stained the cells with fluorescently labeled antibodies for 15 minutes and then analyzed cell populations by multi-color flow cytometry. Dead cells were excluded by a lower level of forward scatter found at the bottom left corner of the density plot according to the guidelines for the use of flow cytometry and cell sorting in immunological studies (32). For OX40 and PD-1 expression analysis, heparinized whole blood samples were stained for 15 minutes with the antibodies and red blood cells were lysed with FACS Lysing Solution (BD Biosciences). The antibodies used for this study are shown in **Supplementary Table 2**. The definition of each CD4+ T cell subsets is shown in **Supplementary Table 3**.

For intracellular staining for IL-21 or surface staining for PD-L1, PBMCs were freshly purified using gradient centrifugation with a Ficoll-Paque Plus (GE Healthcare). CD4+ T cells were enriched from PBMCs using a CD4+ T cell isolation kit (Miltenyi Biotec) according to the manufacturer’s instructions, and then cultured in 96-well round-bottom plates in RPMI 1640 medium supplemented with 10% heat-inactivated fetal bovine serum. The cells were maintained in a humidified atmosphere at 37°C with 5% CO2. Cells (2×10^5^/200 μL/well) were stimulated for 4 hours with phorbol myristoyl acetate (PMA) (Sigma, St. Louis) [50 ng/ml] and ionomycin (Sigma, St. Louis) [1 µg/ml] along with Golgistop (BD Biosciences) [4 μl of BD GolgiStop™ for every 6 mL of cell culture]. Following stimulation, CD4+ T cells were stained for 30 minutes with antibodies specific for cell-surface markers for Tfh and Th cell subsets and BV510- conjugated anti-human PD-L1 (Clone 29E.2A3; BioLegend) (**Supplementary Table 3**). Cells were then fixed and permeabilized with Intracellular Fixation & Permeabilization Buffer Set (eBioscience) according to the manufacturer’s protocol, and then incubated with APC-conjugated anti-human IL-21 (Clone 3A3-N2; BioLegend) for 40 minutes. Cells were then washed and analyzed using a LSRFortessa X-20 (BD Biosciences) flow cytometer. Data were analyzed using FlowJo v.10 software (Tree Star, Stanford University, CA, USA). Single cell mapping was performed using a t-Distributed Stochastic Neighbor Embedding (tSNE) algorithm (33). Gating strategies of flow cytometry data are shown in **Supplementary Figures 1–5**.

### RNA-Sequencing Analysis

Publically available RNA-sequencing data (GSE152904; GSM4629190, GSM4629187, GSM4629184, and GSM4629181) were used for analysis of comprehensive cytokine expression profiles in human CD4+ T cells induced by OX40 signaling (GEO accession). CD4+ T cells were stimulated *in vitro* in the presence of anti-CD3/CD28 activation beads, with or without agonistic OX40 antibody for 3 to 5 days, after which RNA-sequencing was performed to evaluate OX40-mediated transcriptional changes (34). Gene expression was quantified by RSEM (v1.2.14).

### Statistical Analysis

Data were analyzed using GraphPad Prism software (Version7; GraphPad Software, La Jolla, CA). Statistical significance was assessed by unpaired or paired 2-tailed Student t-test or repeated measures ANOVA. Two-sided P < 0.05 was considered significant.

## Results

### Peripheral Tfh Cells Show High Expression of TIGIT

**Figure 1:**
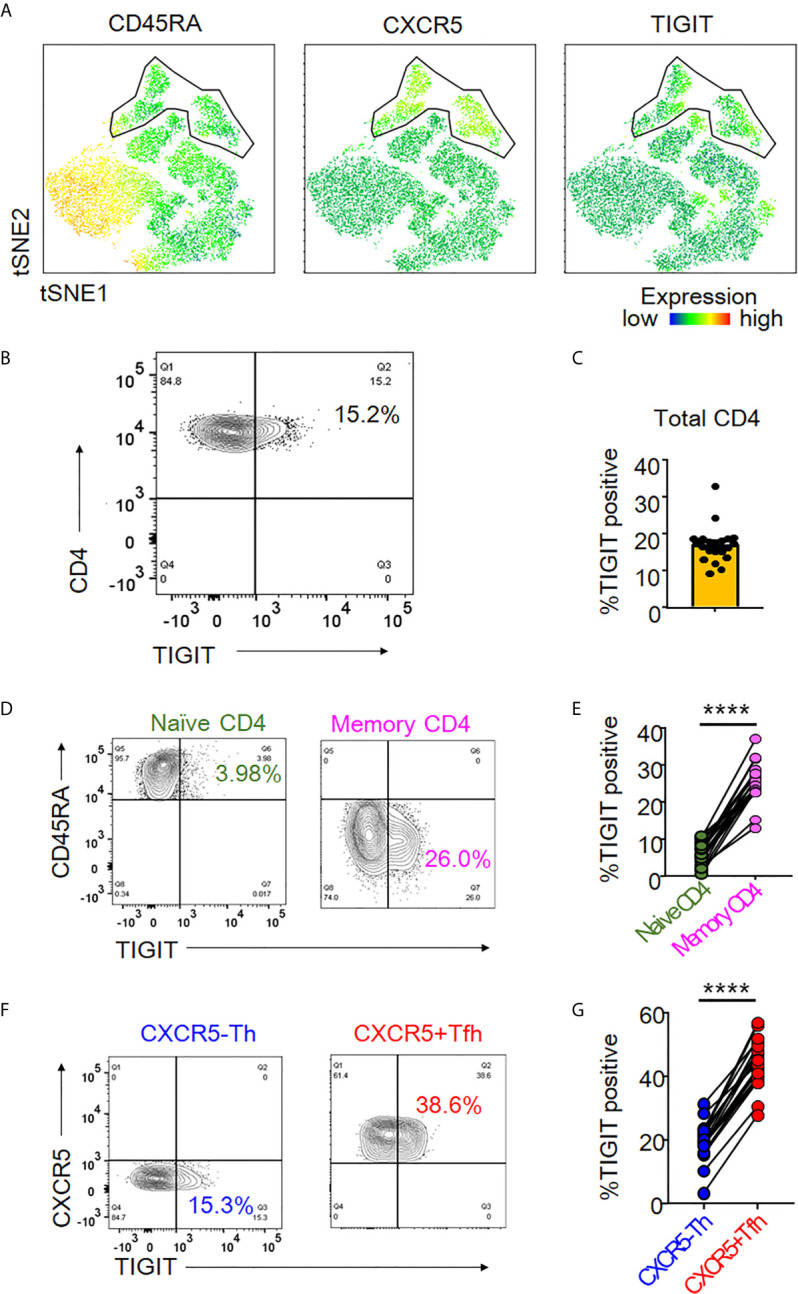
TIGIT expression in peripheral Tfh cells. **(A)** Single cells of CD3^+^CD4^+^ T cell types were plotted by t-Distributed Stochastic Neighbor Embedding (tSNE). Heat maps show expression levels of labeled markers (CD45RA, CXCR5, and TIGIT). Enclosure shows CD45RA-CXCR5+T follicular helper (Tfh) cell population. **(B, C)** The proportion of TIGIT^+^ cells was analyzed in total CD4^+^ T cells from healthy individuals by flow cytometry. **(A)**, representative image. **(B)**, multiple analyses of the proportion of TIGIT^+^ cells in total CD4^+^ T cells (n=21). **(D, E)** Proportion of TIGIT^+^ cells was analyzed in naïve (CD45RA^+^) and memory (CD45RA^-^) CD4^+^ T cells. **(D)**, representative image. **(E)**, multiple comparison of the proportion of TIGIT^+^ cells in naïve and memory CD4^+^ T cells (n=21). **(F, G)** Proportion of TIGIT ^+^ cells was examined in CXCR5^-^CD45RA^-^CD4^+^ T helper cells (CXCR5^-^Th) and CXCR5^+^CD45RA^-^CD4^+^ Tfh (CXCR5^+^ Tfh) cells. **(F)**, representative image. **(G)**, multiple comparisons of the proportion of TIGIT^+^ or TIGIT^-^ cells in CXCR5^-^Th and CXCR5^+^Tfh cells (n=21). **(D, G)** paired t-test. ****p<0.0001.

TIGIT is an immune checkpoint receptor whose expression profile in peripheral CD4+ T cells is not understood in detail. We sought to elucidate its expression profile by first analyzing TIGIT expression in peripheral CD4+ T cells using unbiased tSNE algorithm (33). We found that TIGIT expression mainly came from a CD45RA-CXCR5+CD4+ T cell population, so-called peripheral Tfh cells (**Figure 1A**). To confirm the relevance of TIGIT expression in Tfh cells, we next comprehensively quantified the proportion of TIGIT+ cells in CD4+ T cell subsets from healthy individuals. We found that approximately 20% of total CD4+ T cells were positive for TIGIT (**Figures 1B, C**) and that most TIGIT+ CD4+ T cells belonged to the memory fraction (mean 25% versus. 6%, p <0.0001) (**Figures 1D, E**). We then examined TIGIT expression in memory CD4+ T cells classified into CXCR5+ Tfh and CXCR5- T helper (Th) fractions. We found that the proportion of TIGIT+ cells in the Tfh fraction was twice as high as that in the Th fraction (mean 43% versus. 19%, p <0.0001) (**Figures 1F, G**). These findings suggested that TIGIT was mainly expressed on peripheral Tfh cells.

### TIGIT Identifies Peripheral Tfh-Cell Populations Producing High Levels of IL-21

**Figure 2:**
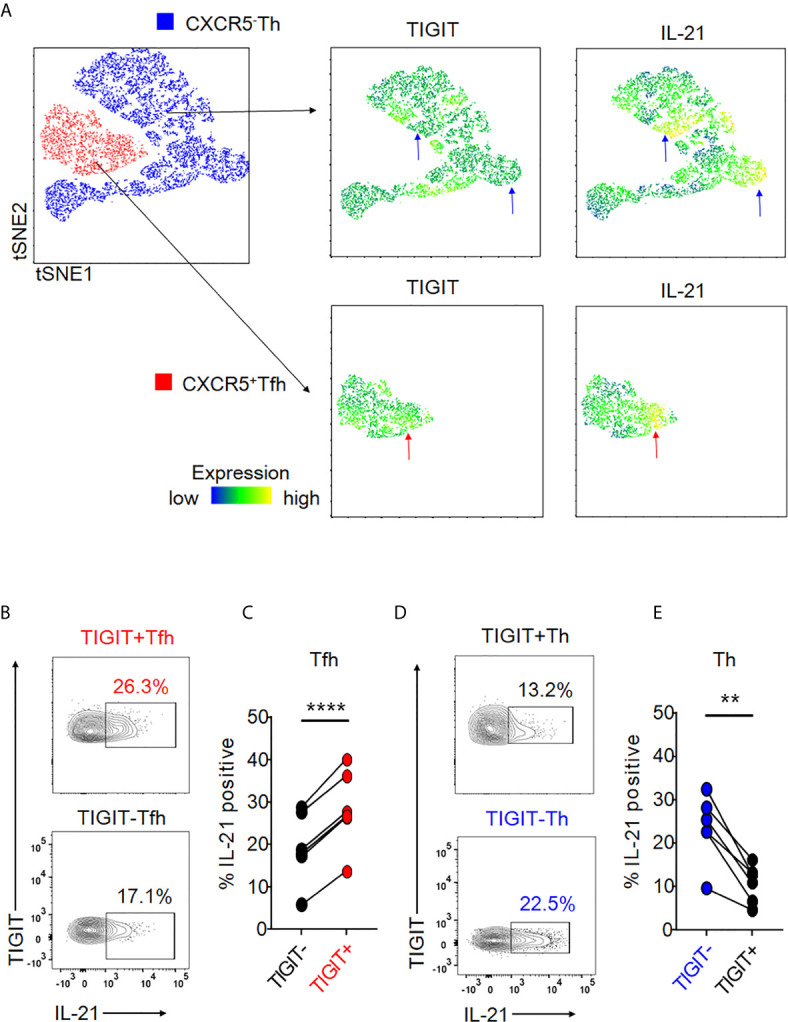
Expression of TIGIT is differentially associated with high IL-21-producing populations in Tfh and Th cells. **(A)** Single cells in Tfh and Th fractions were plotted by tSNE. Heat maps show expression levels of labeled markers (TIGIT and IL-21). Blue arrows indicate that TIGIT^-^Th cells express high levels of IL-21. Red arrows show that TIGIT ^+^Tfh cells express high levels of IL-21. **(B–E)** CD4^+^ T cells were stimulated for 4 hours with phorbol myristoyl acetate and ionomycin. The proportion of intracellular IL-21 production in TIGIT^+^ or TIGIT^-^Tfh and TIGIT^+^Th or TIGIT^-^Th cells was measured by flow cytometry. **(B, D)**, representative image. **(C, E)**, multiple comparison of the proportion of IL-21-producing cells (n=6). **(C, E)** paired t-test. ****p<0.0001, **p<0.01.

Given that peripheral CXCR5+ Tfh cells produce IL-21 (23, 24), we assessed the association of TIGIT expression with IL-21 production in peripheral Tfh cells by comparing IL-21 production in TIGIT+ Tfh and TIGIT- Tfh cells. Unbiased single cell analysis using the tSNE algorithm demonstrated that TIGIT+ Tfh cells showed high expression of IL-21 (**Figure 2A**). Unexpectedly, the null hypothesis was confirmed in Th cells regarding the association between TIGIT expression and IL-21; TIGIT- Th cells showed high expression of IL-21 (**Figure 2A**). To confirm these results, we compared the proportion of IL-21-producing cells between TIGIT+ and TIGIT- Tfh cells from healthy individuals *in vitro*. The results showed that peripheral TIGIT+ Tfh cells produced more IL-21 than TIGIT- Tfh cells (mean 28% versus 19%, p <0.0001) (**Figures 2B, C**). Conversely, when we compared IL-21 production between TIGIT+ Th and TIGIT- Th cells, TIGIT- Th cells produced more IL-21 than TIGIT+ Th cells (mean 23% versus 11%, p <0.01) (**Figures 2D, E**). Thus, peripheral Tfh cells are characterized by high expression of TIGIT, and TIGIT positivity identifies high IL-21-producing Tfh-cell populations. In contrast, the majority of peripheral Th cells do not express TIGIT and TIGIT negativity detects high IL-21-producing Th-cell populations.

### TIGIT Is a Useful Marker for Detecting High IL-21-Producing Populations of Tfh1, Tfh2, and Tfh17 Subsets

**Figure 3:**
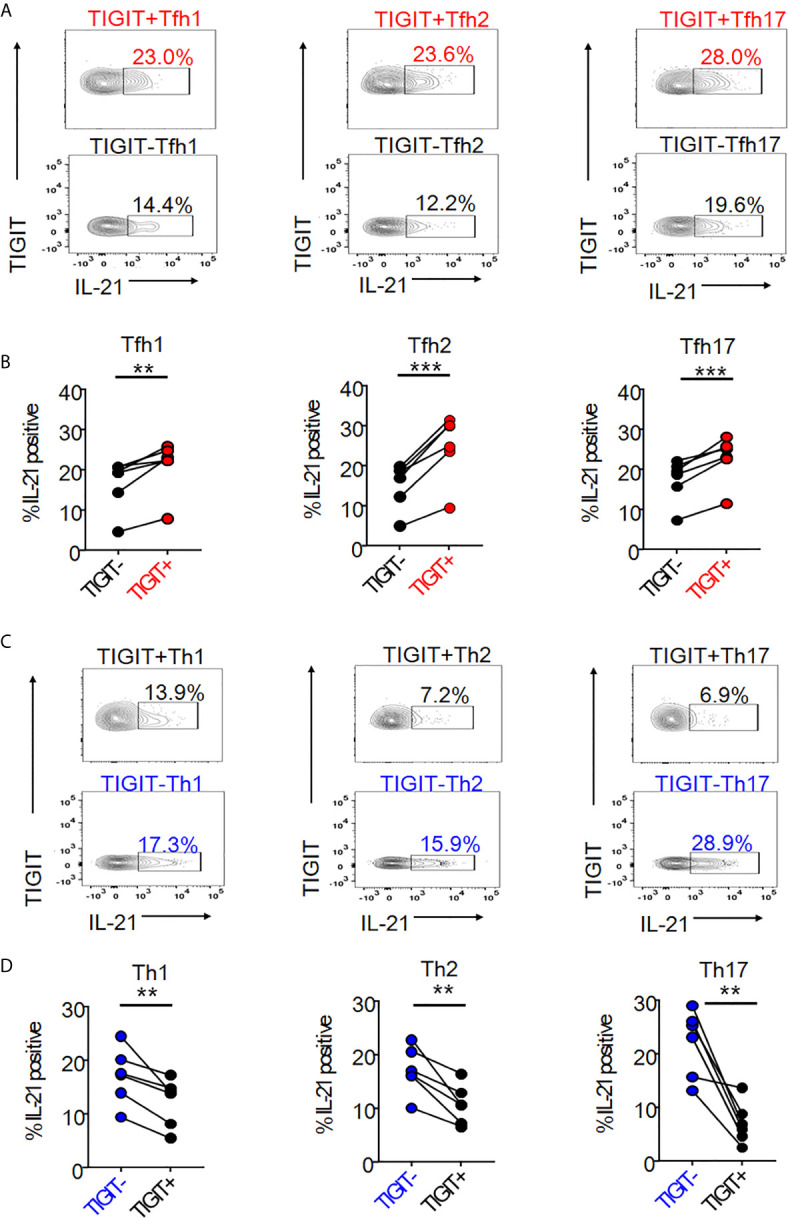
The association of TIGIT expression with IL-21 in Tfh and Th subsets. **(A–D)** CD4^+^ T cells were stimulated for 4 hours with phorbol myristoyl acetate and ionomycin. Intracellular IL-21 production in TIGIT^+^ or TIGIT^-^Tfh cell subsets (Tfh1; CXCR3^+^CCR6^-^ cells, Tfh2; CXCR3^-^CCR6^-^ cells, Tfh17; CXCR3^-^CCR6^+^ cells) or TIGIT^+^ or TIGIT^-^Th cell subsets (Th1; CXCR3^+^CCR6^-^ cells, Th2; CXCR3^-^CCR6^-^ cells, Th17; CXCR3^-^CCR6^-^ cells) were measured by flow cytometry. **(C, E)**, representative image. **(B, D)** multiple comparison of the proportion of IL-21 producing cells in Tfh cell subsets or Th cell subsets (n=6). **(B, D)** paired t-test. ***p<0.001, **p<0.01.

We next hypothesized that TIGIT could be a common surface marker for the detection of high IL-21-producing populations in peripheral Tfh subsets, such as Tfh1, Tfh2, and Tfh17. Single cell mapping of each Tfh and Th cell subset revealed that TIGIT+ Tfh subsets and TIGIT- Th subsets consistently expressed high levels of IL-21 (**Supplementary Figure 6**). Accordingly, we quantified and compared IL-21 production in respective TIGIT+ Tfh and TIGIT- Tfh subsets. TIGIT positivity commonly revealed high IL-21-producing populations in Tfh1 (21% versus 17%, p <0.01), Tfh2 (25% versus 15%, p <0.001), and Tfh17 (23% versus 17%, p <0.001) subsets (**Figures 3A, B**). Consistent with this result for high IL-21-producing TIGIT- Th cell subsets by single cell analysis, TIGIT negativity commonly detected high IL-21-producing populations in Th1 (17% versus 12%, p <0.01), Th2 (17% versus 11%, p <0.01), and Th17 (22% versus 7%, p <0.01) subsets (**Figures 3C, D**). Thus, although chemokine receptor programming on human Th cells can be plastic (35), TIGIT is a stable marker for detecting IL-21-producing populations in Tfh and Th cells.

### TIGIT Is Not an Activation Marker

**Figure 4:**
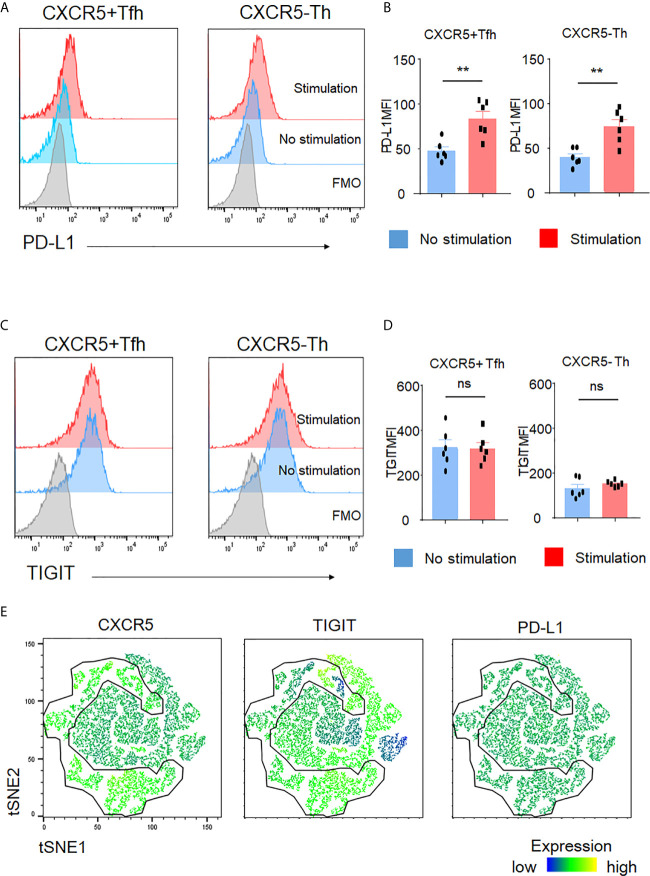
TIGIT is not an activation marker. **(A–D)** CD4^+^ T cells were stimulated for 4 hours with phorbol myristoyl acetate and ionomycin. Surface expression of PD-L1 and TIGIT in Tfh cells or Th cells was measured by flow cytometry. **(A, C)**, representative image. **(B, D)**, multiple comparison results (n=6). FMO, Fluorescence minus one control. **(E)** Stimulated single cell was plotted by tSNE and expression levels of labeled markers (CXCR5, TIGIT, and PD-L1) are shown as a heatmap. **(B, D)** paired t-test. **p<0.01, ns p>0.05.

Activated T cells generally produce more cytokines than non-activated T cells. Thus, we asked whether TIGIT was an activation marker for CD4+ T cells. To do so, we assayed PD-L1, a recently recognized activation marker for T cells (36). After stimulation, we observed significant upregulation of PD-L1 in both Tfh and Th cells (**Figures 4A, B**). In contrast, TIGIT expression in Tfh and Th cells did not show significant changes (**Figures 4C, D**). To further test that TIGIT was not an activation marker, we used PD-L1 expression levels as activation markers and compared them between TIGIT+ Tfh and TIGIT- Tfh cells after stimulation. The results clearly showed that there was no difference in PD-L1 expression levels between TIGIT+ and TIGIT- cells from Tfh cells (**Figure 4E**). These data suggest that TIGIT is not an activation marker, but rather identifies high IL-21-producing populations of Tfh cells.

### PD-1 Expression Is Higher in TIGIT+ T Cells Than TIGIT- T Cells

**Figure 5:**
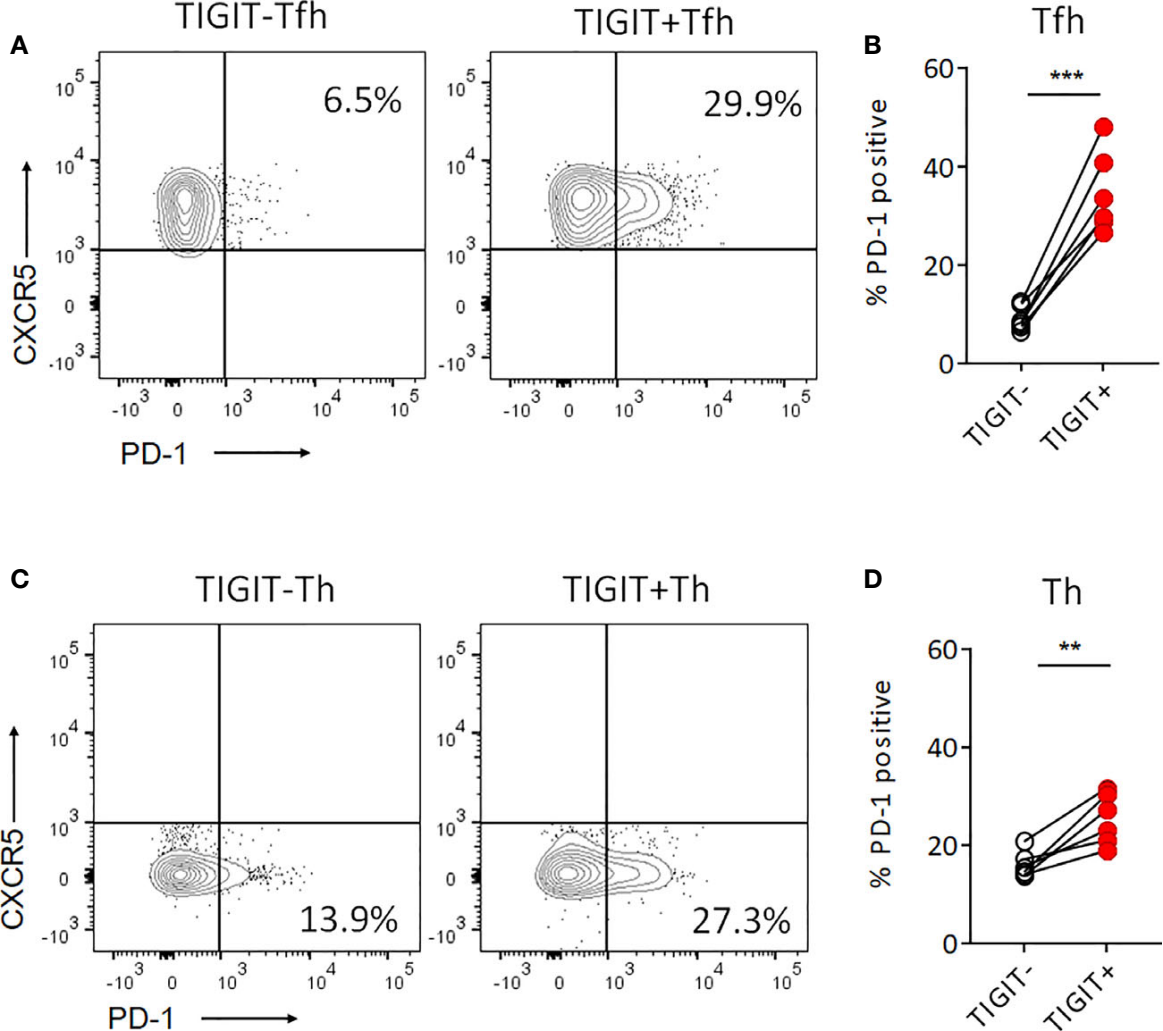
Proportion of PD-1^+^cells is higher in TIGIT^+^ T cells than TIGIT^-^ T cells. **(A, B)** PD-1 expression level was examined in TIGIT^+^Tfh and TIGIT^-^Tfh cells by flow cytometry. **(C, D)** PD-1 expression level was examined in TIGIT^+^Th and TIGIT^-^Th cells by flow cytometry. **(A, C)**, representative image. **(B, D)**, multiple comparison results (n=6). **(B, D)** paired t-test. ***p<0.001, ***p<0.01.

Recent studies reported that PD-1+ CXCR5- Th cells, so-called T peripheral helper (Tph) cells, effectively produced IL-21 (37–39). Also, PD-1+ CXCR5+ Tfh cells are known to be high IL-21 producers (40). Thus, we next investigated the proportion of PD-1+ cells in TIGIT+ and TIGIT- T cell fractions. TIGIT+ Tfh cells showed higher proportion of PD-1+ cells than TIGIT- Tfh cells (34.7% versus 9.2%, p <0.001) (**Figures 5A, B**). As well, TIGIT+ Th cells had higher proportion of PD-1+ cells than TIGIT- Th cells (25.4% versus 16.1%, p <0.01) (**Figures 5C, D**). These results indicate that high IL-21 production in TIGIT+ Tfh cells may be explained by high proportion of PD-1+ cells in their fraction; however, TIGIT+ Th cells contained more Tph than TIGIT- Th cells, suggesting that some other mechanisms rather than Tph cells might be contributing to high IL-21 production in TIGIT- Th cells.

### OX40 Signal Is Associated With High IL-21 Expression

**Figure 6:**
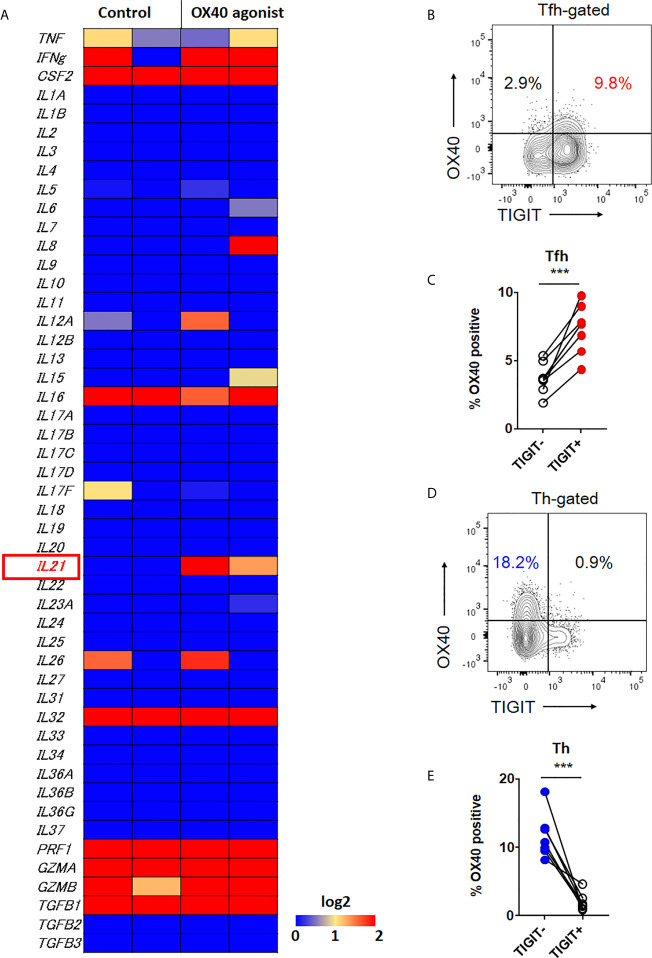
OX40 signal is associated with IL-21 expression. **(A)** CD4^+^ T cells were stimulated *in vitro* in the presence of αCD3/CD28 activation beads with or without agonistic OX40 antibody for 3 to 5 days. Transcriptome analysis for 49 cytokines was obtained by RNA-sequencing, and the gene expression levels are shown as a heatmap. **(B, D)** OX40 expression level was examined in CXCR5+Tfh and CXCR5-Th cells by flow cytometry. **(B, D)**, representative image. **(C, E)**, multiple comparison results (n=7). **(C, E)** paired t-test. ***p<0.001.

Therefore, we further investigated other molecule which could contribute to high IL-21 production in both TIGIT+ Tfh cells and TIGIT- Th cells. Recently, it has been suggested that OX40 (CD134)-mediated signal is a key contributor to Tfh-associated responses (41–43). RNA-sequencing analysis revealed that among a total of 49 cytokines, IL-21 expression was clearly induced by OX40 stimulation in CD4+ T cells (**Figure 6A**). Accordingly, we assumed that TIGIT+ Tfh and TIGIT- Th cells express higher levels of OX40. Flow cytometric analysis found that the proportion of OX40+ cells was higher in TIGIT+ Tfh cells than TIGIT- Tfh cells (7.3% versus 3.7%, p <0.001) (**Figures 6B, C**). In contrast, TIGIT- Th cells showed a higher proportion of OX40+ cells than TIGIT+ Th cells (11.8% versus 2.0%, p <0.001) (**Figures 6D, E**). Taken together, these data suggested that TIGIT+ Tfh and TIGIT- Th cells exhibit high IL-21 production *via* OX40-mediated signal.

### Peripheral TIGIT+ Tfh Populations Are Increased in IgG4-RD

The identification of high IL-21-producing peripheral TIGIT+ Tfh-cell populations led us to consider whether TIGIT expression in Tfh cells represents accelerated Tfh responses in IgG4-RD. IgG4-RD is a fibro-inflammatory autoimmune disease in which peripheral Tfh, IL-21, and autoantibodies are involved (44–47).

**Figure 7:**
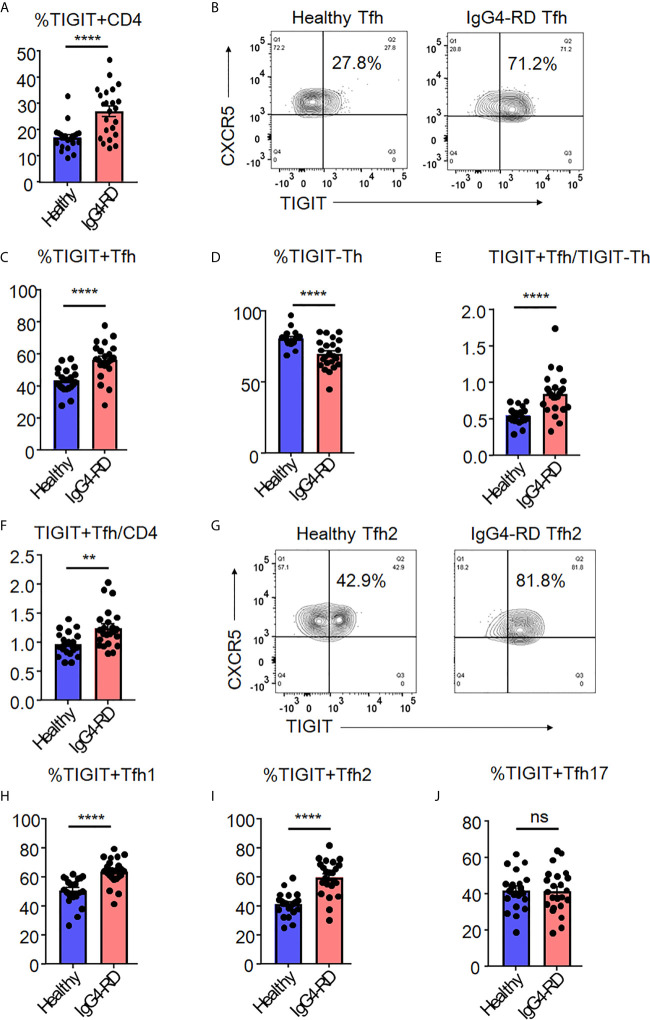
The increase of TIGIT ^+^ Tfh cells in IgG4-RD. The proportion of TIGIT^+^ CD4^+^ T cells **(A)**, TIGIT^+^ Tfh cells **(B, C)**, TIGIT^-^ Th cells **(D)**, the ratio of TIGIT^+^ Tfh to TIGIT^-^ Th cells **(E)**, and the ratio of TIGIT^+^ Tfh to CD4^+^ T cells **(F)** were examined in blood from patients with untreated, active IgG4-RD (n=23) or healthy individuals (n=21). The proportion of TIGIT^+^ Tfh1 **(H)**, TIGIT^+^ Tfh2 **(G, I)**, and TIGIT^+^ Tfh17 **(J)** cells was analyzed and compared. **(A, C–F, H–J)** unpaired t test. ****p<0.0001, **p<0.01, ns p>0.05.

We analyzed the proportion of peripheral high IL-21-producing populations (TIGIT+ Tfh cells and TIGIT- Th cells) in samples from patients with untreated, active IgG4-RD compared to those from healthy individuals. When we examined TIGIT+ cells in total CD4+ T cells, IgG4-RD patients showed higher proportion of TIGIT+ cells than healthy individuals (mean 27% versus 17%, p<0.0001) (**Figure 7A**). Furthermore, we found that the proportion of TIGIT+ Tfh cells was significantly higher in IgG4-RD patients than in healthy individuals (mean 56% versus 43%, p<0.0001) (**Figures 7B, C**). We also found that the proportion of TIGIT- Th cells was significantly lower in IgG4-RD patients than in healthy individuals (mean 70% versus 80%, p<0.001) (**Figure 7D**). Accordingly, as compared with healthy individuals, the balance of peripheral TIGIT+ Tfh and TIGIT- Th cells was skewed toward TIGIT+ Tfh cells in IgG4-RD (mean 0.84 versus 0.55, p<0.001) (**Figure 7E**). The increased ratio of TIGIT+ Tfh cells to CD4 ^+^ T cells or to TIGIT- Tfh cells in IgG4-RD compared to healthy individuals was also confirmed (**Figure 7F** and **Supplementary Figure 7**).

Since we found significant skewing toward TIGIT+ Tfh cells in patients with IgG4-RD, we next analyzed the proportion of TIGIT+ Tfh-cell subsets (Tfh1, Tfh2, and Tfh17) among IgG4-RD and healthy individuals (**Figures 7G–J**). The proportion of peripheral TIGIT+ Tfh1 or TIGIT+ Tfh2 cells in IgG4-RD was significantly higher than that in healthy individuals, whereas the proportion of peripheral TIGIT+ Tfh17 cells was not different between in IgG4-RD and healthy individuals (**Figures 7G–J**).

The proportion of peripheral TIGIT+ Tfh cells thus appears to be increased in IgG4-RD in which Tfh cells are involved; moreover, the balance of peripheral TIGIT+ Tfh subset (Tfh1, Tfh2, and Tfh17) is characteristically altered. IgG4-RD patients showed an increased proportion of peripheral TIGIT+ Tfh cells, particularly in Tfh1 and Tfh2 populations, compared with healthy individuals.

### The Increases in the Proportion of Peripheral TIGIT+ Tfh Cells Are Associated With Disease Activity in IgG4-RD

**Figure 8:**
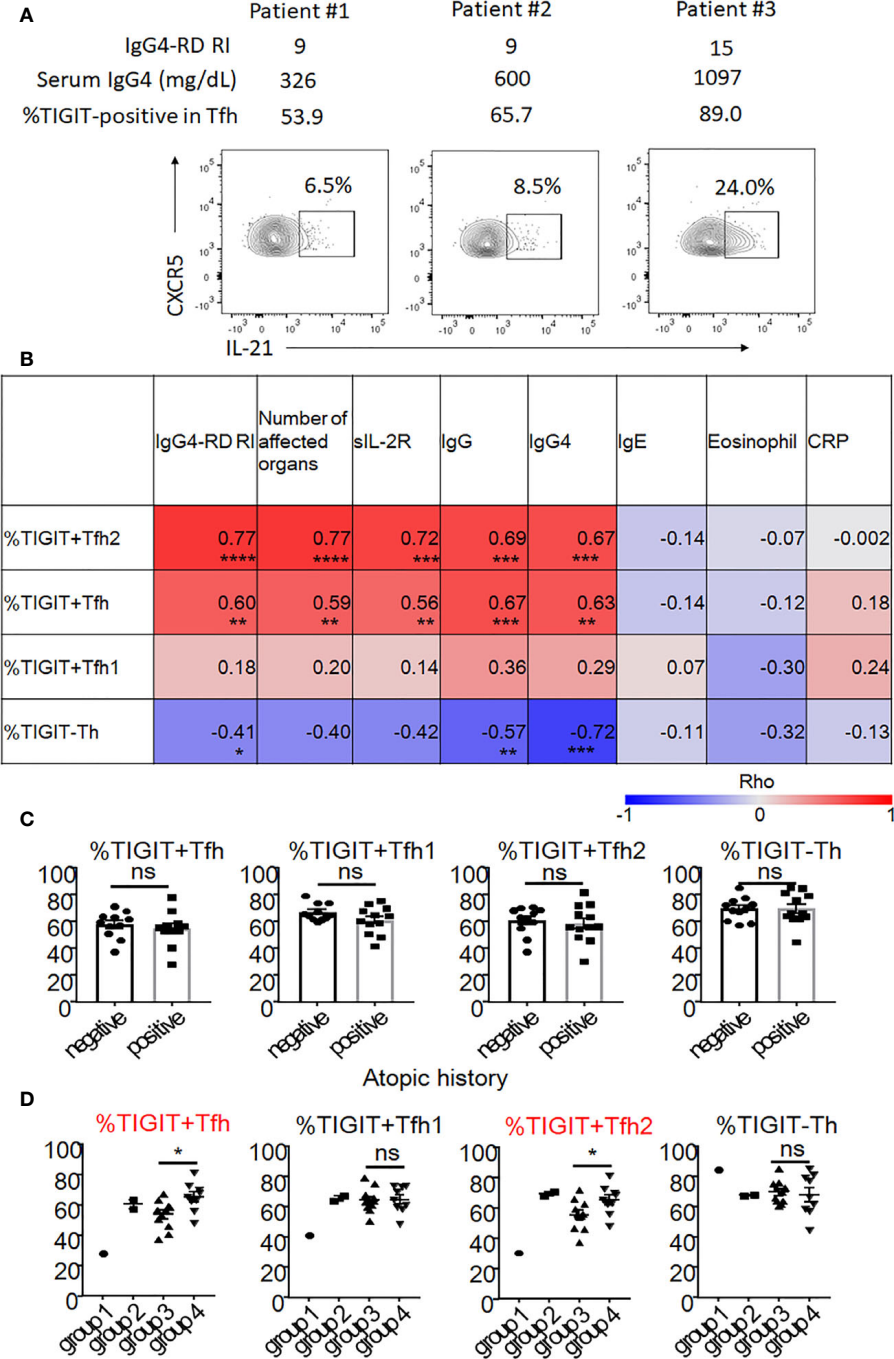
The association of proportion of TIGIT^+^Tfh cells with disease activity and history of atopy of IgG4-RD. Correlation of IL-21 production in Tfh with disease activity was examined in 3 patients with untreated, active IgG4-RD **(A)**. Correlation of TIGIT^+^ Tfh, TIGIT^+^ Tfh1, TIGIT^+^ Tfh2, and TIGIT^-^ Th with clinical parameters in 23 patients with active, untreated IgG4-RD are shown as heat maps **(B)**. The proportion of TIGIT^+^ Tfh, TIGIT^+^ Tfh1, TIGIT^+^ Tfh2, and TIGIT^-^ Th was compared between patients with an atopic history and those without **(C)**. The proportion of TIGIT^+^ Tfh, TIGIT^+^ Tfh1, TIGIT^+^ Tfh2, and TIGIT^-^ Th are shown based on each clinical phenotype (groups 1-4). Statistical comparison was performed on groups 3 and 4 **(D)**. **(B)** Spearman’s correlation coefficient. **(C, D)** unpaired t test. ***p<0.0001, ***p<0.001, **p<0.01, *p<0.05, ns p>0.05.

To determine the clinical significance of increased numbers of peripheral TIGIT+ Tfh cells and their subsets, we determined the correlation between these cells and clinical parameters in untreated, active patients. First, we demonstrated the correlation between IL-21 production in Tfh cells and disease activity in patients with active, untreated IgG4-RD (n = 3, **Figure 8A**). As a result, we found that IL-21 production in Tfh cells correlated with the proportion of TIGIT+ cells in Tfh cells, serum IgG4 level, and scores of disease activity even though the significance was not determined. Further analysis of 23 patients with active, untreated IgG4-RD demonstrated that the proportion of TIGIT+ Tfh cells correlated with the scores of IgG4-RD RI, number of affected organs and soluble IL-2 receptors, and serum levels of IgG and IgG4 (**Figure 8B**). The proportion of TIGIT+ Tfh2 cells also correlated with these clinical parameters; this correlation was stronger than that of TIGIT+ Tfh cells (**Figure 8B**). In contrast, the proportion of TIGIT- Th cells negatively correlated with the scores of IgG4-RD RI and serum levels of IgG and IgG4 (**Figure 8B**). There was no significant correlation between the proportion of TIGIT+ Tfh1 cells and clinical parameters (**Figure 8B**).

Atopic parameters (serum IgE level or blood eosinophil count) or serum C-reactive protein levels did not correlate with the proportion of any immune cell subsets in IgG4-RD (**Figure 8B**). Consistent with this finding, the increase of peripheral TIGIT+ Tfh cells in IgG4-RD did not depend on the atopic history of patients (**Figure 8C**).

Recent cross-sectional studies in international cohorts have revealed that IgG4-RD patients can be divided into 4 groups based on clinical phenotype: group 1, Pancreato-Hepato-Biliary disease; group 2, Retroperitoneal Fibrosis and/or Aortitis; group 3, Head and Neck-limited disease; and group 4, classic Mikulicz syndrome with systemic involvement (48). We correlated the proportion of peripheral TIGIT+ Tfh, TIGIT+ Tfh1, TIGIT+ Tfh2, and TIGIT- Th cells with these clinical groupings. We compared the proportion of these cell subsets between groups 3 and 4 (**Figure 8D**). Groups 1 and 2 could not be evaluated because of small patient numbers (1 for G1 and 2 for G2). Compared with group 3, group 4 had a higher proportion of TIGIT+ Tfh cells (mean 65% versus 54%, p<0.05) and TIGIT+ Tfh2 cells (mean 65% versus 56%, p<0.05), but not of TIGIT+ Tfh1 cells (mean 65% versus 65%, p=0.92) or TIGIT- Th cells (mean 68% versus 70%, p=0.72) (**Figure 8D**). Group 4 also had higher disease activity scores (mean 15 versus 10, p<0.01) and levels of serum IgG (mean 2120mg/dL versus 1551mg/dL, p<0.01) (**Supplementary Figure 8**), and serum IgG4 level tended to be higher in group 4 than group 3 (752mg/dL versus 440mg/dL, p=0.07) (**Supplementary Figure 8**). Collectively, the increased proportion of peripheral TIGIT+ Tfh and TIGIT+ Tfh2 cells reflected higher disease activity in patients with untreated, active IgG4-RD.

### Peripheral TIGIT+ Tfh Cells in IgG4-RD Are Decreased Following Treatment With Glucocorticoids

**Figure 9:**
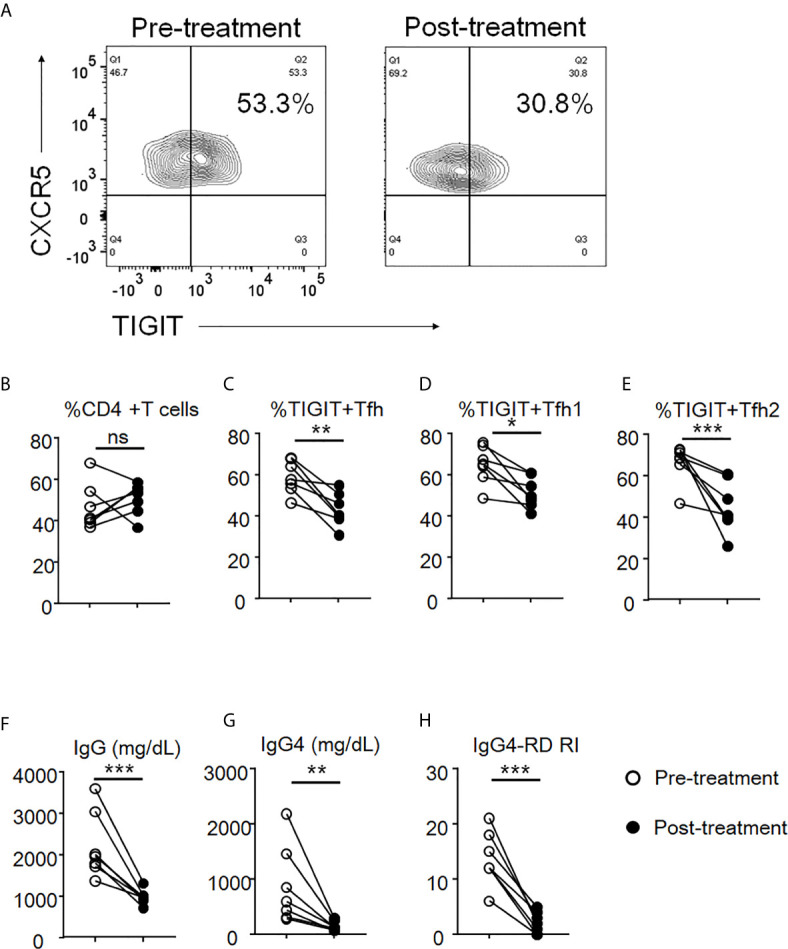
The decrease of proportion of TIGIT^+^ Tfh cells after glucocorticoid treatment. The proportion of total CD4 + T cells **(B)**, TIGIT+ Tfh **(A, C)**, TIGIT+ Tfh1 **(D)**, and TIGIT+ Tfh2 **(E)** were examined after 12 weeks of glucocorticoid treatment. Serum levels of IgG **(F)** and IgG4 **(G)** as well as the scores of IgG4-RD RI **(H)** were also analyzed. n = 7. **(B, E)** and **(F, G)** repeated measures ANOVA. **(H)** paired t-test. ***p<0.001, **p<0.01, *p<0.05, ns p>0.05.

To evaluate the effects of glucocorticoid treatment on the proportions of peripheral TIGIT+ Tfh, TIGIT+ Tfh1, and TIGIT+ Tfh2 cells and on clinical improvement, we evaluated seven patients with IgG4-RD before and after 12 weeks of glucocorticoid treatment. The proportion of peripheral TIGIT+ Tfh, TIGIT+ Tfh1, and TIGIT+ Tfh2 cells significantly decreased after glucocorticoid treatment (TIGIT+ Tfh, 59% versus 43%, p<0.01; TIGIT+ Tfh1, 65% versus 51%, p<0.05; and TIGIT+ Tfh2, 66% versus 45%, p<0.01), while the proportion of total CD4 + T cells did not significantly decrease (47% versus 51%, p>0.05) (**Figures 9A–E**). Serum IgG and IgG4 levels and disease activity scores also decreased (mean 2209mg/dL versus 980mg/dL, p<0.01; mean 868mg/dL versus 147mg/dL, p<0.05; and mean 14 versus 2, p<0.001, respectively) (**Figures 9F–H**).

## Discussion

In this study, we found that peripheral Tfh cells have higher expression of TIGIT than Th cells. Of note, TIGIT+ Tfh cells produced more IL-21 than TIGIT- Tfh cells. TIGIT positivity commonly identified high IL-21-producing populations on analysis of Tfh subsets (Tfh1, Tfh2, and Tfh17). OX40 signal was associated with high IL-21 expression, and TIGIT+ Tfh cells exhibited higher OX40 expression than TIGIT- Tfh cells. Importantly, the proportion of peripheral TIGIT+ Tfh and TIGIT+ Tfh2 cells was increased in patients with IgG4-RD, correlating with disease activity. These findings suggest that OX40 is associated with high IL-21 production in TIGIT+ Tfh cells and the increase of TIGIT+ Tfh cells reflects disease activity in IgG4-RD.

TIGIT positivity was associated with high production of IL-21 in peripheral Tfh cells, whereas TIGIT negativity was linked to high production of IL-21 in peripheral Th cells. The biological significance that there is a positive correlation of IL-21 production in Tfh but not Th cells might be derived from OX40 but not Tph cells. In fact, the proportion of Tph in TIGIT- Th cells was lower than in TIGIT+ Th cells, whereas both TIGIT+ Tfh and TIGIT- Th cells showed high expression of OX40. Furthermore, we showed that OX40 signal was associated with high IL-21 expression. Thus, we believe that OX40 signal contributes to high IL-21 production in TIGIT+ Tfh and TIGIT- Th cells. Immune checkpoint and co-stimulatory signals play a critical role in regulating immune responses in the context of autoimmunity and cancer (49). Considering the role of TIGIT as a co-inhibitory receptor and the excessive formation of IL-21-induced tertiary lymphoid organs in the disease lesions of autoimmune diseases (50), targeting TIGIT might represent a novel therapeutic approach to the restraint of aberrant Tfh responses (51). Our study also suggests that blocking OX40 signal might also be an attractive therapeutic target.

Among the four distinct clinical phenotypes reported recently, we found that group 4 exhibited higher proportions of peripheral TIGIT+ Tfh cells than group 3, along with higher disease activity. Thus, we assume that IL-21-producing cells are associated with disease activity and the pathogenesis of IgG4-RD. The low number of patients in groups 1 (Pancreato-Hepato-Biliary disease; n=1) and group 2 (Retroperitoneal Fibrosis and/or Aortitis; n=2) were insufficient to support statistical analysis. Nevertheless, we did make the interesting observation that both patients in group 2 had extremely high levels of TIGIT+ Tfh2 cells (68% and 71%) compared to healthy individuals (41%). The patient comprising group 1 had lower levels of TIGIT+ Tfh2 cells (30%) than healthy individuals. Accordingly, differential dysregulation of peripheral Tfh subsets responses might correlate with the different clinical phenotypes observed. Further studies are needed to support this idea.

Our present study has the limitation. We did not show the localization of TIGIT+ Tfh cells in the lesions of patients with IgG4-RD. Recent studies reported that Tfh and Tfh2 cells are infiltrated in the affected tissues of IgG4-RD (6, 9, 14). The co-localization of Tfh cells with B cells within ectopic germinal centers at the lesions of IgG4-RD was found (52). Furthermore, Maehara et al. reported that the infiltration of IL-4-producing Tfh cells presented outside of the ectopic germinal centers at lesions of IgG4-RD (10), while IL-21-producing Tfh cells presented inside of the ectopic germinal centers (20), indicating that IL-21-producing TIGIT+ Tfh cells may localize inside the ectopic germinal centers interacting with B cells.

In conclusion, OX40 is associated with high IL-21-producing peripheral TIGIT+ Tfh-cell populations, and the increase in TIGIT+ Tfh cells reflects disease activity in IgG4-RD. Our findings suggest that TIGIT may be a useful marker for detecting IL-21-producing Tfh cells and a potential therapeutic target.

## Data Availability Statement

The datasets presented in this study can be found in online repositories. The names of the repository/repositories and accession number(s) can be found in the article/**Supplementary Material**.

## Ethics Statement

The studies involving human participants were reviewed and approved by Keio University School of Medicine. The patients/participants provided their written informed consent to participate in this study.

## Author Contributions

Study design: MA. Data acquisition: MA, KS, KY, HY, YK, and TT. Data analysis and interpretation: MA, KS, and KY. Manuscript drafting: MA, KS, KY, and TT. All authors contributed to the article and approved the submitted version.

## Funding

This research did not receive any specific grant from funding agencies in the public, commercial, or not-for-profit sectors.

## Conflict of Interest

The authors declare that the research was conducted in the absence of any commercial or financial relationships that could be construed as a potential conflict of interest.

## References

[1] Perugino CA Stone JH. IgG4-related disease: an update on pathophysiology and implications for clinical care. Nat Rev Rheumatol (2020) 16:702–14. 10.1038/s41584-020-0500-7 32939060

[2] Lanzillotta M Mancuso G Della-Torre E. Advances in the diagnosis and management of IgG4 related disease. BMJ (2020) 369:m1067. 10.1136/bmj.m1067 32546500

[3] Akiyama M Takeuchi T. IgG4-Related Disease: Beyond Glucocorticoids. Drugs Aging (2018) 35:275–87. 10.1007/s40266-018-0534-6 29546609

[4] Akiyama M Kaneko Y Takeuchi T. Characteristics and prognosis of IgG4-related periaortitis/periarteritis: A systematic literature review. Autoimmun Rev (2019) 18:102354. 10.1016/j.autrev.2019.102354 31323364

[5] Akiyama M Suzuki K Yasuoka H Kaneko Y Yamaoka K Takeuchi T. Follicular helper T cells in the pathogenesis of IgG4-related disease. Rheumatol (Oxford) (2018) 57:236–45. 10.1093/rheumatology/kex171 28460058

[6] Kamekura R Takano K Yamamoto M Kawata K Shigehara K Jitsukawa S. Cutting Edge: A Critical Role of Lesional T Follicular Helper Cells in the Pathogenesis of IgG4-Related Disease. J Immunol (2017) 199:2624–9. 10.4049/jimmunol.1601507 28916523

[7] Kubo S Nakayamada S Zhao J Yoshikawa M Miyazaki Y Nawata A. Correlation of T follicular helper cells and plasmablasts with the development of organ involvement in patients with IgG4-related disease. Rheumatol (Oxford) (2018) 57:514–24. 10.1093/rheumatology/kex455 29253269

[8] Grados A Ebbo M Piperoglou C Groh M Regent A Samson M. T Cell Polarization toward T H 2/T FH 2 and T H 17/T FH 17 in Patients with IgG4-Related Disease. Front Immunol (2017) 8:235. 10.3389/fimmu.2017.00235 28348556 PMC5347096

[9] Chen Y Lin W Yang H Wang M Zhang P Feng R. Aberrant Expansion and Function of Follicular Helper T Cell Subsets in IgG4-Related Disease. Arthritis Rheumatol (2018) 70:1853–65. 10.1002/art.40556 PMC6220938 29781221

[10] Maehara T Mattoo H Mahajan VS Murphy SJ Yuen GJ Ishiguro N. The expansion in lymphoid organs of IL-4 + BATF + T follicular helper cells is linked to IgG4 class switching in vivo. Life Sci Alliance (2018) 1:e201800050. 10.26508/lsa.201800050 29984361 PMC6034714

[11] Akiyama M Suzuki K Yamaoka K Yasuoka H Takeshita M Kaneko Y. Number of Circulating Follicular Helper 2 T Cells Correlates With IgG4 and Interleukin-4 Levels and Plasmablast Numbers in IgG4-Related Disease. Arthritis Rheumatol (2015) 67:2476–81. 10.1002/art.39209 25989153

[12] Akiyama M Yasuoka H Yamaoka K Suzuki K Kaneko Y Kondo H. Enhanced IgG4 production by follicular helper 2 T cells and the involvement of follicular helper 1 T cells in the pathogenesis of IgG4-related disease. Arthritis Res Ther (2016) 18:167. 10.1186/s13075-016-1064-4 27411315 PMC4944254

[13] Higashioka K Ota Y Maehara T Moriyama M Ayano M Mitoma H. Association of circulating SLAMF7 + Tfh1 cells with IgG4 levels in patients with IgG4-related disease. BMC Immunol (2020) 21:31. 10.1186/s12865-020-00361-0 32487061 PMC7268355

[14] Cargill T Makuch M Sadler R Lighaam LC Peters R van Ham M. Activated T-Follicular Helper 2 Cells Are Associated With Disease Activity in IgG4-Related Sclerosing Cholangitis and Pancreatitis. Clin Transl Gastroenterol (2019) 10:e00020. 10.14309/ctg.0000000000000020 31033594 PMC6602789

[15] Takanashi S Akiyama M Suzuki K Otomo K Takeuchi T. IgG4-related fibrosing mediastinitis diagnosed with computed tomography-guided percutaneous needle biopsy: Two case reports and a review of the literature. Med (Baltimore) (2018) 97:e10935. 10.1097/MD.0000000000010935 PMC6393095 29851832

[16] Mattoo H Mahajan VS Maehara T Deshpande V Della-Torre E Wallace ZS. Clonal expansion of CD4(+) cytotoxic T lymphocytes in patients with IgG4-related disease. J Allergy Clin Immunol (2016) 138:825–38. 10.1016/j.jaci.2015.12.1330 PMC5014627 26971690

[17] Maehara T Mattoo H Ohta M Mahajan VS Moriyama M Yamauchi M. Lesional CD4+ IFN-γ+ cytotoxic T lymphocytes in IgG4-related dacryoadenitis and sialoadenitis. Ann Rheum Dis (2017) 76:377–85. 10.1136/annrheumdis-2016-209139 PMC5435236 27358392

[18] Della-Torre E Bozzalla-Cassione E Sciorati C Ruggiero E Lanzillotta M Bonfiglio S. A CD8α- Subset of CD4+SLAMF7+ Cytotoxic T Cells Is Expanded in Patients With IgG4-Related Disease and Decreases Following Glucocorticoid Treatment. Arthritis Rheumatol (2018) 70:1133–43. 10.1002/art.40469 PMC6019645 29499100

[19] Yabe H Kamekura R Yamamoto M Murayama K Kamiya S Ikegami I. Cytotoxic Tph-like cells are involved in persistent tissue damage in IgG4-related disease. Mod Rheumatol (2021) 31:249–60. 10.1080/14397595.2020.1719576 32023137

[20] Maehara T Moriyama M Nakashima H Miyake K Hayashida JN Tanaka A. Interleukin-21 contributes to germinal centre formation and immunoglobulin G4 production in IgG4-related dacryoadenitis and sialoadenitis, so-called Mikulicz’s disease. Ann Rheum Dis (2012) 71:2011–19. 10.1136/annrheumdis-2012-201477 22753386

[21] Spolski R Leonard WJ. Interleukin-21: a double-edged sword with therapeutic potential. Nat Rev Drug Discovery (2014) 13:379–95. 10.1038/nrd4296 24751819

[22] Long D Chen Y Wu H Zhao M Lu Q. Clinical significance and immunobiology of IL-21 in autoimmunity. J Autoimmun (2019) 99:1–14. 10.1016/j.jaut.2019.01.013 30773373

[23] Ueno H. Human Circulating T Follicular Helper Cell Subsets in Health and Disease. J Clin Immunol (2016) 36:34–9. 10.1007/s10875-016-0268-3 26984851

[24] Morita R Schmitt N Bentebibel SE Ranganathan R Bourdery L Zurawski G. Human blood CXCR5(+)CD4(+) T cells are counterparts of T follicular cells and contain specific subsets that differentially support antibody secretion. Immunity (2011) 34:108–21. 10.1016/j.immuni.2010.12.012 PMC3046815 21215658

[25] Gorvel L Olive D. Targeting the “PVR-TIGIT axis” with immune checkpoint therapies. F1000Res (2020) 9:F1000 Faculty Rev–354. 10.12688/f1000research.22877.1 PMC7222031 32489646

[26] Banga R Rebecchini C Procopio FA Noto A Munoz O Ioannidou K. Lymph node migratory dendritic cells modulate HIV-1 transcription through PD-1 engagement. PloS Pathog (2019) 15:e1007918. 10.1371/journal.ppat.1007918 31329640 PMC6675123

[27] Seth S Ravens I Kremmer E Maier MK Hadis U Hardtke S. Abundance of follicular helper T cells in Peyer’s patches is modulated by CD155. Eur J Immunol (2009) 39:3160–70. 10.1002/eji.200939470 19688744

[28] Umehara H Okazaki K Masaki Y Kawano M Yamamoto M Saeki T. Comprehensive diagnostic criteria for IgG4-related disease (IgG4-RD), 2011. Mod Rheumatol (2012) 22:21–30. 10.3109/s10165-011-0571-z 22218969

[29] Wallace ZS Naden RP Chari S Choi HK Della-Torre E Dicaire JF. The 2019 American College of Rheumatology/European League Against Rheumatism classification criteria for IgG4-related disease. Ann Rheum Dis (2020) 79:77–87. 10.1136/annrheumdis-2019-216561 31796497

[30] Wallace ZS Naden RP Chari S Choi H Della-Torre E Dicaire JF. The 2019 American College of Rheumatology/European League Against Rheumatism Classification Criteria for IgG4-Related Disease. Arthritis Rheumatol (2020) 72:7–19. 10.1002/art.41120 31793250

[31] Carruthers MN Stone JH Deshpande V Khosroshahi A. Development of an IgG4-RD Responder Index. Int J Rheumatol (2012) 2012:259408. 10.1155/2012/259408 22611406 PMC3348627

[32] Cossarizza A Chang HD Radbruch A Akdis M Andrä I Annunziato F. Guidelines for the use of flow cytometry and cell sorting in immunological studies. Eur J Immunol (2017) 47:1584–797. 10.1002/eji.201646632 PMC9165548 29023707

[33] Amir el AD Davis KL Tadmor MD Simonds EF Levine JH Bendall SC. viSNE enables visualization of high dimensional single-cell data and reveals phenotypic heterogeneity of leukemia. Nat Biotechnol (2013) 31:545–52. 10.1038/nbt.2594 PMC4076922 23685480

[34] Campos Carrascosa L van Beek AA de Ruiter V Doukas M Wei J Fisher TS. FcγRIIB engagement drives agonistic activity of Fc-engineered αOX40 antibody to stimulate human tumor-infiltrating T cells. J Immunother Cancer (2020) 8:e000816. 10.1136/jitc-2020-000816 32900860 PMC7478034

[35] Sallusto F. Heterogeneity of Human CD4(+) T Cells Against Microbes. Annu Rev Immunol (2016) 34:317–34. 10.1146/annurev-immunol-032414-112056 27168241

[36] Diskin B Adam S Cassini MF Sanchez G Liria M Aykut B. PD-L1 engagement on T cells promotes self-tolerance and suppression of neighboring macrophages and effector T cells in cancer. Nat Immunol (2020) 21:442–54. 10.1038/s41590-020-0620-x 32152508

[37] Bocharnikov AV Keegan J Wacleche VS Cao Y Fonseka CY Wang G. PD-1hiCXCR5- T peripheral helper cells promote B cell responses in lupus via MAF and IL-21. JCI Insight (2019) 4:e130062. 10.1172/jci.insight.130062 PMC6824311 31536480

[38] Rao DA. T Cells That Help B Cells in Chronically Inflamed Tissues. Front Immunol (2018) 9:1924. 10.3389/fimmu.2018.01924 30190721 PMC6115497

[39] Rao DA Gurish MF Marshall JL Slowikowski K Fonseka CY Liu Y. Pathologically expanded peripheral T helper cell subset drives B cells in rheumatoid arthritis. Nature (2017) 542:110–4. 10.1038/nature20810 PMC5349321 28150777

[40] Ueno H. T follicular helper cells in human autoimmunity. Curr Opin Immunol (2016) 43:24–31. 10.1016/j.coi.2016.08.003 27588918

[41] Kurata I Matsumoto I Ohyama A Osada A Ebe H Kawaguchi H. Potential involvement of OX40 in the regulation of autoantibody sialylation in arthritis. Ann Rheum Dis (2019) 78:1488–96. 10.1136/annrheumdis-2019-215195 31300460

[42] Pattarini L Trichot C Bogiatzi S Grandclaudon M Meller S Keuylian Z. TSLP-activated dendritic cells induce human T follicular helper cell differentiation through OX40-ligand. J Exp Med (2017) 214:1529–46. 10.1084/jem.20150402 PMC5413322 28428203

[43] Jacquemin C Schmitt N Contin-Bordes C Liu Y Narayanan P Seneschal J. OX40 Ligand Contributes to Human Lupus Pathogenesis by Promoting T Follicular Helper Response. Immunity (2015) 42:1159–70. 10.1016/j.immuni.2015.05.012 PMC4570857 26070486

[44] Perugino CA AlSalem SB Mattoo H Della-Torre E Mahajan V Ganesh G. Identification of galectin-3 as an autoantigen in patients with IgG4-related disease. J Allergy Clin Immunol (2019) 143:736–745.e6. 10.1016/j.jaci.2018.05.011 29852256 PMC6265117

[45] Liu H Perugino CA Ghebremichael M Wallace ZS Montesi SB Stone JH. Disease Severity Linked to Increase in Autoantibody Diversity in IgG4-Related Disease. Arthritis Rheumatol (2020) 72:687–93. 10.1002/art.41140 PMC7113109 31612628

[46] Hubers LM Vos H Schuurman AR Erken R Oude Elferink RP Burgering B. Annexin A11 is targeted by IgG4 and IgG1 autoantibodies in IgG4-related disease. Gut (2018) 67:728–35. 10.1136/gutjnl-2017-314548 28765476

[47] Shiokawa M Kodama Y Sekiguchi K Kuwada T Tomono T Kuriyama K. Laminin 511 is a target antigen in autoimmune pancreatitis. Sci Transl Med (2018) 10:eaaq0997. 10.1126/scitranslmed.aaq0997 30089633

[48] Wallace ZS Zhang Y Perugino CA Naden R Choi HK Stone JH. Clinical phenotypes of IgG4-related disease: an analysis of two international cross-sectional cohorts. Ann Rheum Dis (2019) 78:406–12. 10.1136/annrheumdis-2018-214603 PMC6996288 30612117

[49] Ramos-Casals M Brahmer JR Callahan MK Flores-Chávez A Keegan N Khamashta MA. Immune-related adverse events of checkpoint inhibitors. Nat Rev Dis Primers (2020) 6:38. 10.1038/s41572-020-0160-6 32382051 PMC9728094

[50] Marinkovic T Marinkovic D. Biological mechanisms of ectopic lymphoid structure formation and their pathophysiological significance. Int Rev Immunol (2020) 7:1–13. 10.3109/08830189009061761 32631119

[51] Dixon KO Schorer M Nevin J Etminan Y Amoozgar Z Kondo T. Functional Anti-TIGIT Antibodies Regulate Development of Autoimmunity and Antitumor Immunity. J Immunol (2018) 200:3000–7. 10.4049/jimmunol.1700407 PMC5893394 29500245

[52] Zaidan M Cervera-Pierot P Seigneux S Dahan K Fabiani B Callard P. Evidence of follicular T-cell implication in a case of IgG4-related systemic disease with interstitial nephritisss. Nephrol Dial Transplant (2011) 26:2047–50. 10.1093/ndt/gfr097 21406542

