# Supplementary material for: Peripheral TIGIT+ T Follicular Helper Cells That Produce High Levels of Interleukin-21 via OX40 Represent Disease Activity in IgG4-Related Disease
Source: Front Immunol. 2021 Apr 14;12:651357. doi: 10.3389/fimmu.2021.651357 (PMC8079782; doi:10.3389/fimmu.2021.651357)
Supplement: Supplementary file 1 [file DataSheet_1.pdf]

Supplementary Table 1. Characteristics of 26 patients with untreated, active IgG4-related disease.

**Demographic features**	
Age, years (median, range)	64 (36-86)
Sex, female (n, %)	12 (52)
Past atopic history (n, %)	12 (52)
Organ involvement (n, %)	
Lacrimal glands and orbits	18 (78)
Salivary glands	14 (61)
Lymph nodes	13 (57)
Lungs	8 (35)
Pancreas	4 (17)
Kidneys	3 (13)
Aorta	2 (9)
Retroperitoneum	2 (9)
Skin	2 (9)
Paravertebral mass	2 (9)
Clinical parameters (median, range)	
Serum IgG (mg/dL)	1734 (934-3593)
Serum IgG4 (mg/dL)	434 (65-2178)
Serum IgE (IU/mL)	310 (5-3300)
Serum C-reactive protein (mg/dL)	0.04 (0.01-3.61)
Serum soluble interleukin-2 receptor (U/mL)	375 (202-1963)
Blood eosinophil count (cells/μL)	252 (44-1568)
Number of affected organs	3 (1-6)
IgG4-RD responder index	12 (6-21)

Supplementary Table 2. Antibodies used for multi-color flow cytometry.

Antibody	Conjugate	Clone	Supplier
CD3	FITC	UCHT1	BD Biosciences
CD4	BV786	SK3	BD Biosciences
CD45RA	BV711	HI100	BD Biosciences
CXCR5	BV421	RF8B2	BD Biosciences
CXCR3	PE	1C6	BD Biosciences
CCR6	PE-CF594	G034E3	BioLegend
TIGIT	BV605	A15153G	BioLegend
PD-L1	BV510	29E.2A3	BioLegend
OX40	APC/Cy7	Ber-ACT35	BioLegend
PD-1	PE	EH12.1	BD Pharmingen™

Supplementary Table 3. Definition of blood $CD4^+$ T cell subsets.

$CD4^+$ T cell subset	Definition
Total $CD4^+$ T cells	$CD3^+CD4^+$ cells
Naïve $CD4^+$ T cells	$CD3^+CD4^+CD45RA^+$ cells
Memory $CD4^+$ T cells	$CD3^+CD4^+CD45RA^-$ cells
T helper (Th) cells	$CD3^+CD4^+CD45RA^-CXCR5^-$ cells
Th1	$CD3^+CD4^+CD45RA^-CXCR5^-CXCR3^+CCR6^-$ cells
Th2	$CD3^+CD4^+CD45RA^-CXCR5^-CXCR3^-CCR6^-$ cells
Th17	$CD3^+CD4^+CD45RA^-CXCR5^-CXCR3^-CCR6^+$ cells
T follicular helper (Tfh) cells	$CD3^+CD4^+CD45RA^-CXCR5^+$ cells
Tfh1	$CD3^+CD4^+CD45RA^-CXCR5^+CXCR3^+CCR6^-$ cells
Tfh2	$CD3^+CD4^+CD45RA^-CXCR5^+CXCR3^-CCR6^-$ cells
Tfh17	$CD3^+CD4^+CD45RA^-CXCR5^+CXCR3^-CCR6^+$ cells

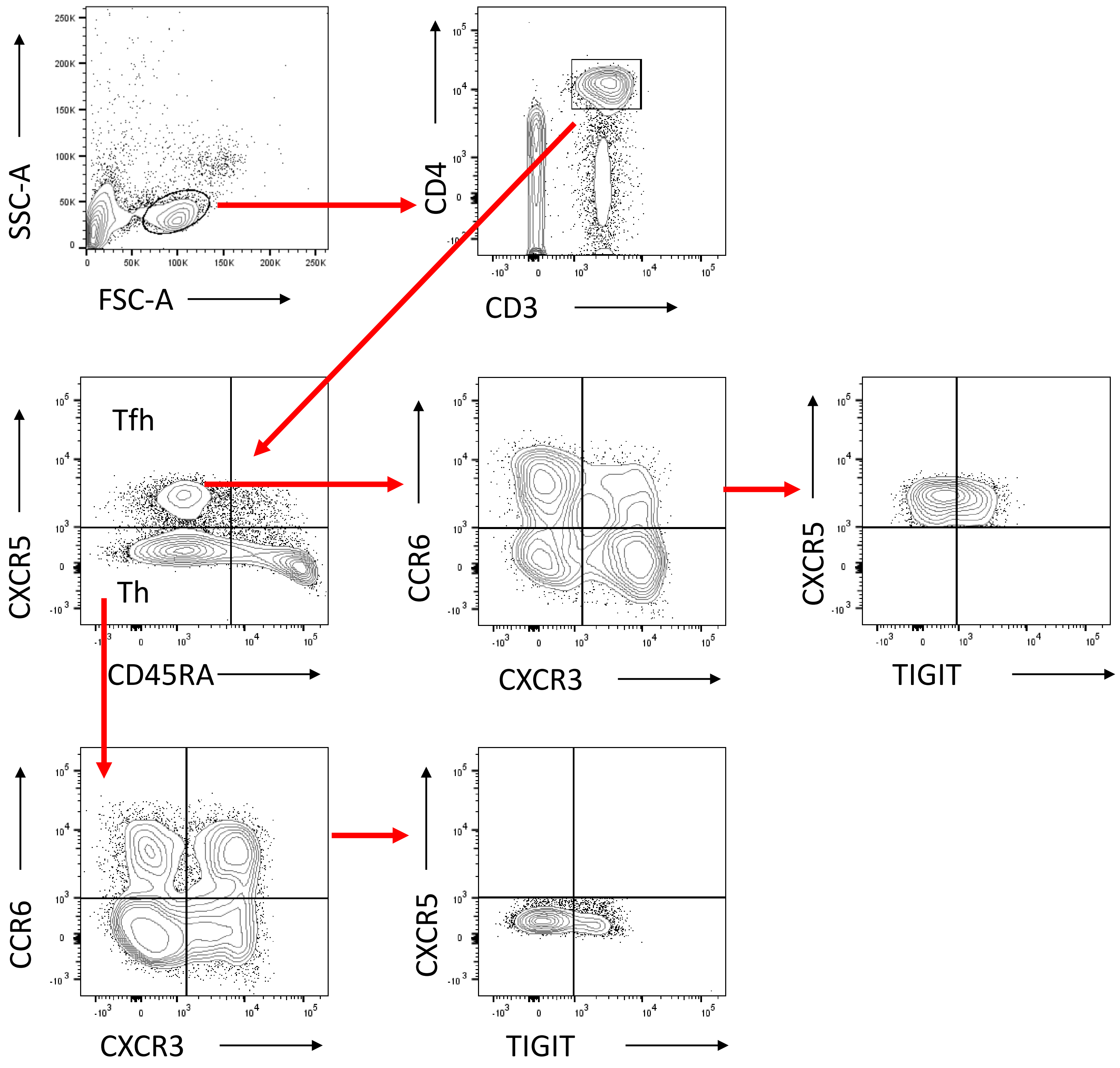

Supplementary Figure 1. Gating strategy for TIGIT+ cells in CD4+T cells.

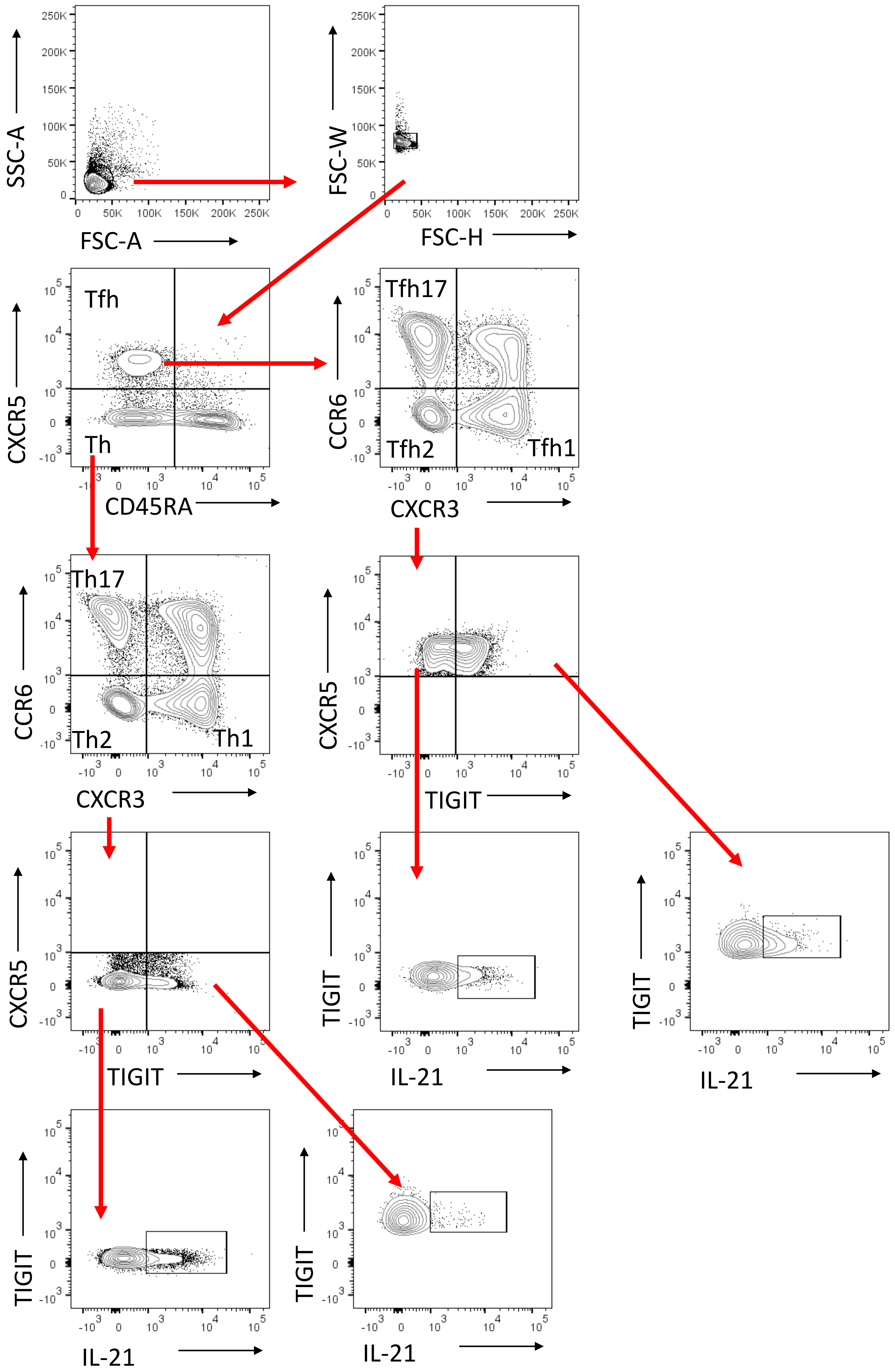

Supplementary Figure 2. Gating strategy for IL-21+ cells in TIGIT+ and TIGIT- CD4+T cells.

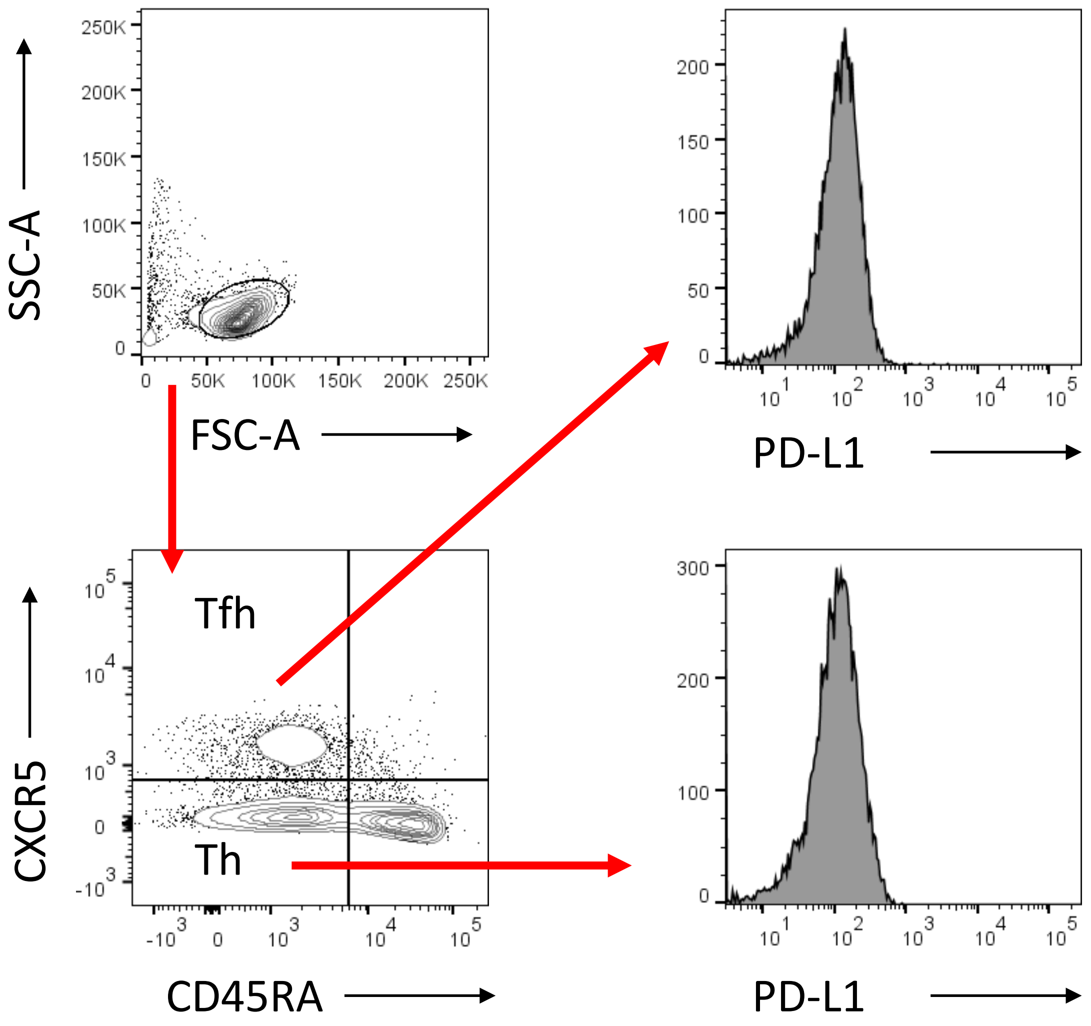

Supplementary Figure 3. Gating strategy for PD-L1 expression in CD4+T cells.

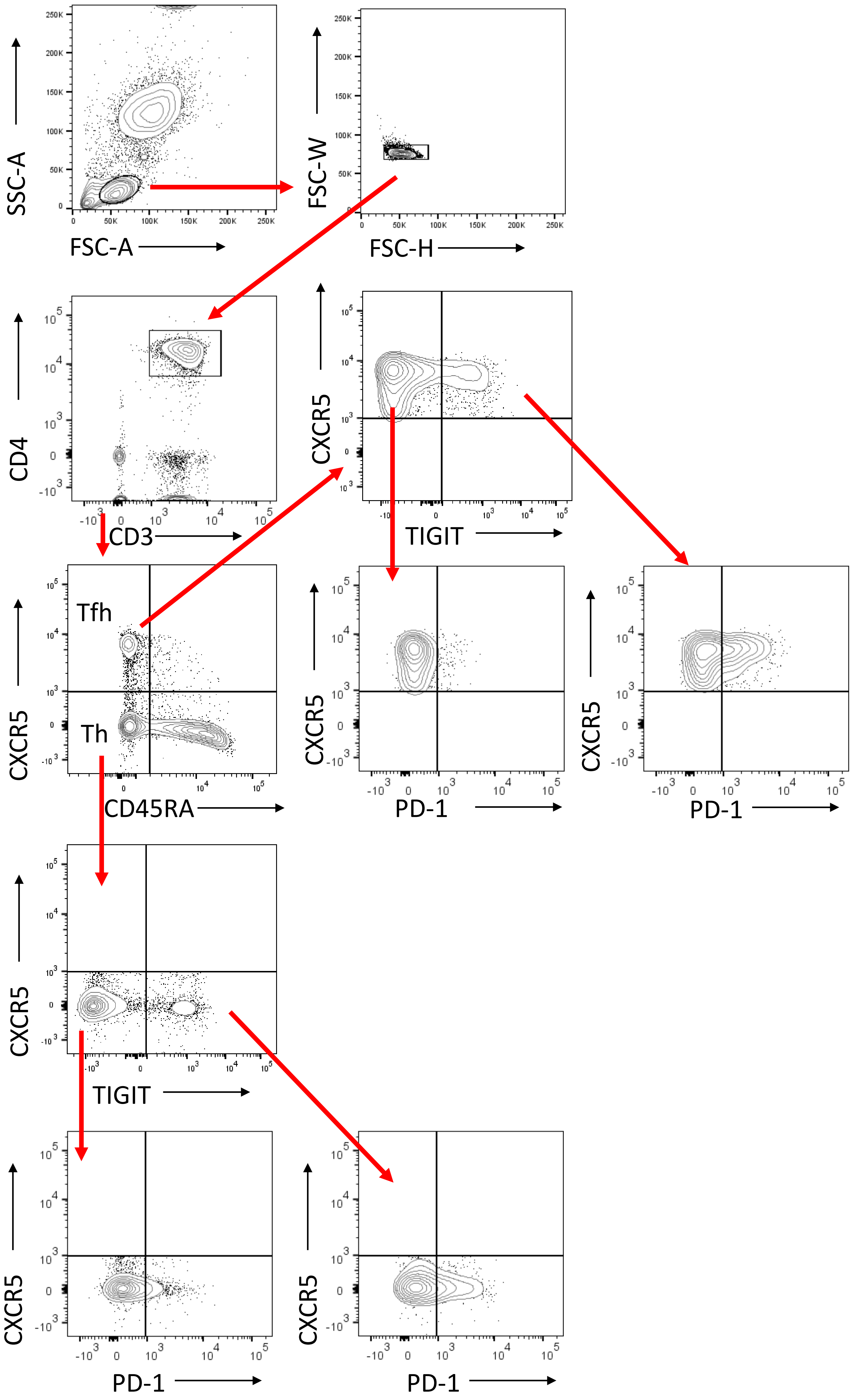

Supplementary Figure 4. Gating strategy for PD-1+ cells in TIGIT+ and TIGIT- CD4+T cells.

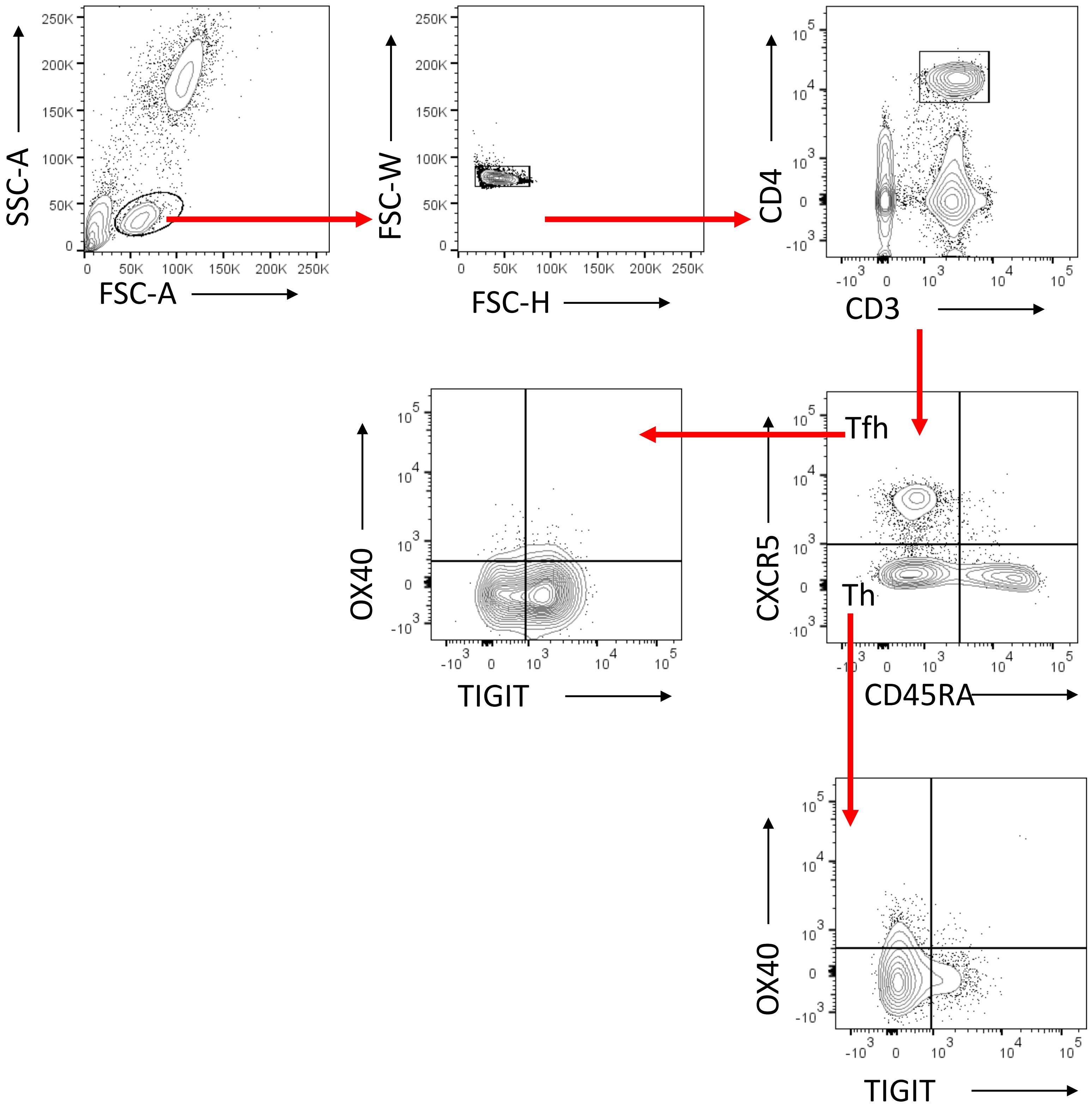

Supplementary Figure 5. Gating strategy for OX40+ cells in TIGIT+ and TIGIT- CD4+T cells.

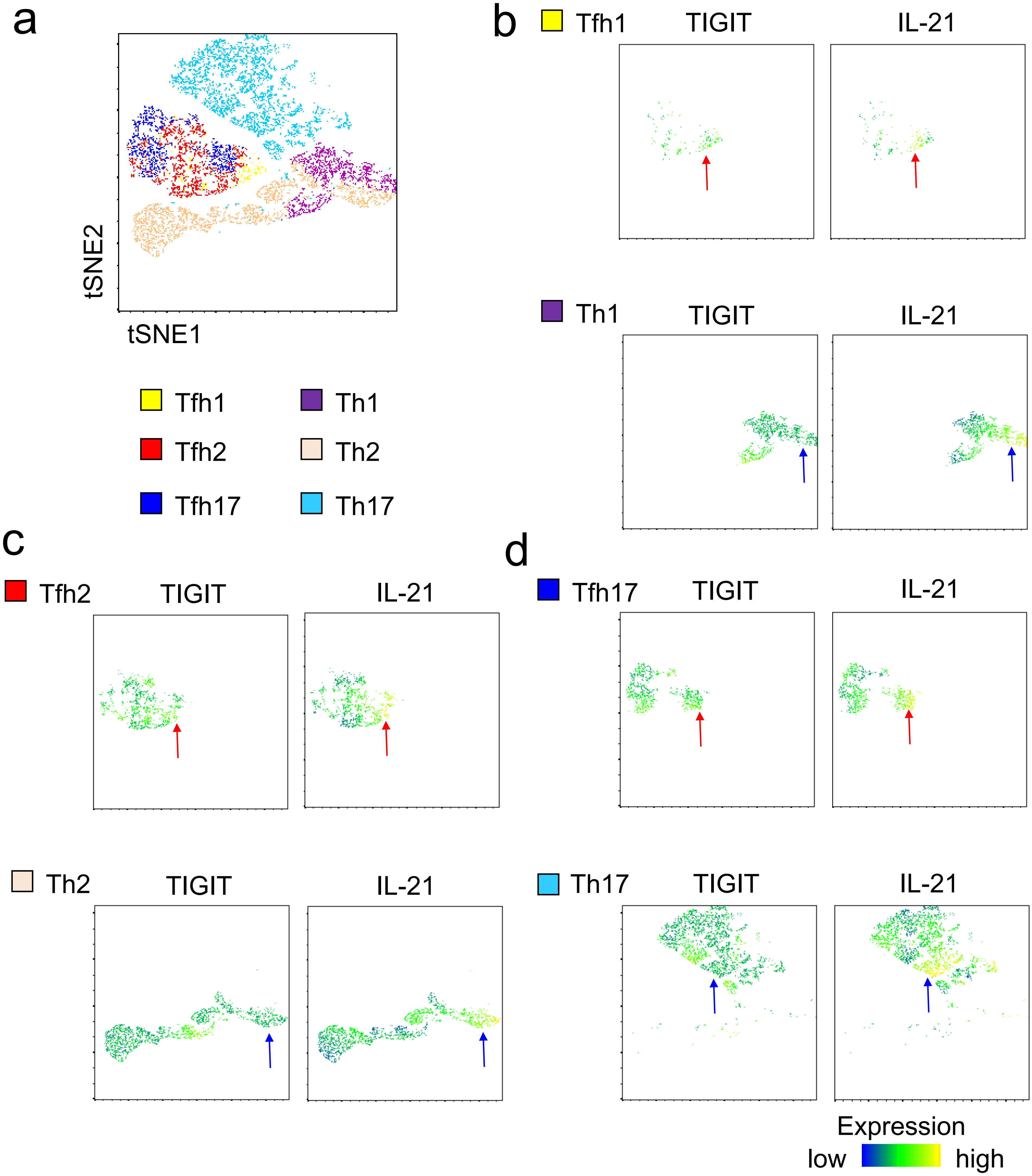

Supplementary Figure 6. Subpopulations of TIGIT-expressing Tfh and Th cells show differential production of IL-21.

(a, b, c, d) Each single cell in Tfh cell subsets (Tfh1, Tfh2, and Tfh17) and Th cell subsets (Th1, Th2, and Th17) was plotted by tSNE. Expression levels of labeled markers (TIGIT and IL-21) are shown as heat maps. Blue arrows indicate that TIGIT^{-}Th subsets express high levels of IL-21. Red arrows indicate that TIGIT^{+}Tfh subsets express high levels of IL-21.

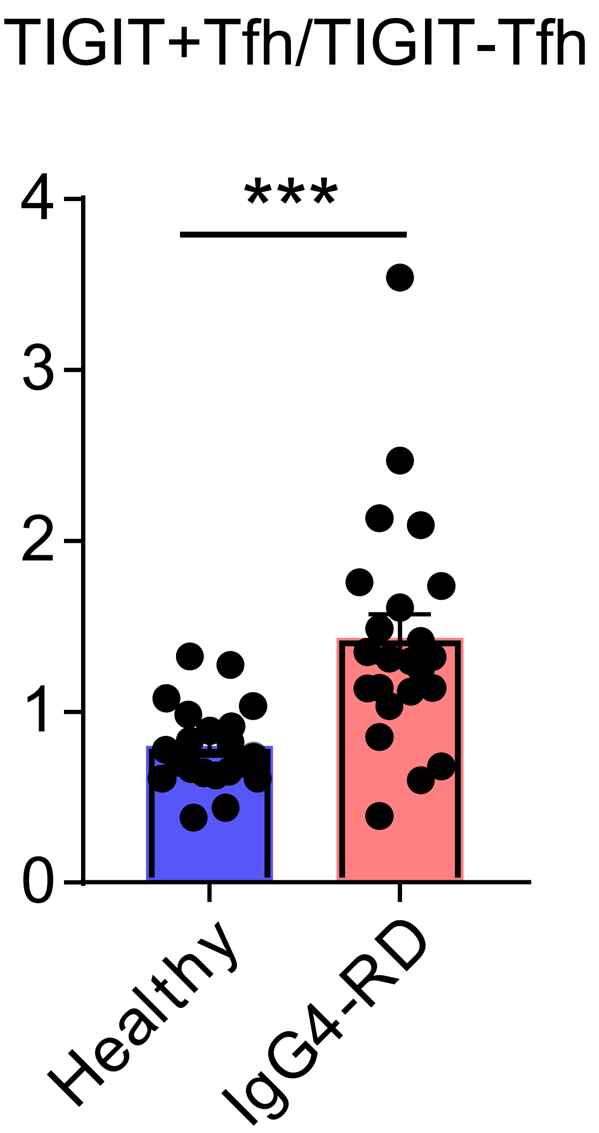

Supplementary Figure 7. The increased ratio of TIGIT+ Tfh to TIGIT- Tfh cells in IgG4-RD.

The ratio of TIGIT+ Tfh to TIGIT- Th cells was examined in blood from patients with untreated, active IgG4-RD (n=23) or healthy individuals (n=21). unpaired t test. *** $p<0.001$.

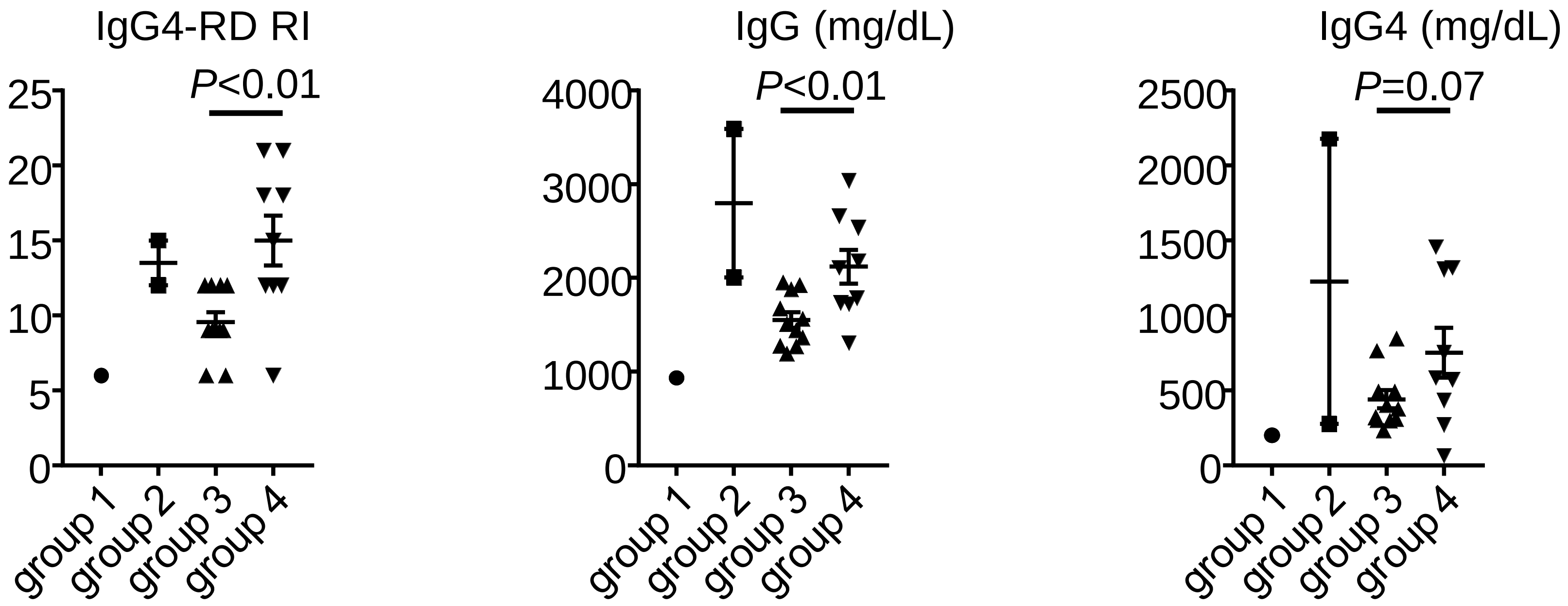

Supplementary Figure 8. Correlation of disease activity score, serum IgG and IgG4 levels with clinical phenotypes.

Scores of IgG4-RD RI and levels of serum IgG and IgG4 are shown relative to clinical phenotype. Statistical analysis was performed for groups 3 and 4.
